# Nanoscale Membrane Domain Formation Driven by Cholesterol

**DOI:** 10.1038/s41598-017-01247-9

**Published:** 2017-04-25

**Authors:** Matti Javanainen, Hector Martinez-Seara, Ilpo Vattulainen

**Affiliations:** 1 Laboratory of Physics, Tampere University of Technology, Tampere, Finland; 2 Department of Physics, University of Helsinki, Helsinki, Finland; 3 Institute of Organic Chemistry and Biochemistry, Czech Academy of Sciences, Prague, Czech Republic; 4 MEMPHYS - Centre for Biomembrane Physics, University of Southern Denmark, Odense, Denmark

## Abstract

Biological membranes generate specific functions through compartmentalized regions such as cholesterol-enriched membrane nanodomains that host selected proteins. Despite the biological significance of nanodomains, details on their structure remain elusive. They cannot be observed via microscopic experimental techniques due to their small size, yet there is also a lack of atomistic simulation models able to describe spontaneous nanodomain formation in sufficiently simple but biologically relevant complex membranes. Here we use atomistic simulations to consider a binary mixture of saturated dipalmitoylphosphatidylcholine and cholesterol — the “minimal standard” for nanodomain formation. The simulations reveal how cholesterol drives the formation of fluid cholesterol-rich nanodomains hosting hexagonally packed cholesterol-poor lipid nanoclusters, both of which show registration between the membrane leaflets. The complex nanodomain substructure forms when cholesterol positions itself in the domain boundary region. Here cholesterol can also readily flip–flop across the membrane. Most importantly, replacing cholesterol with a sterol characterized by a less asymmetric ring region impairs the emergence of nanodomains. The model considered explains a plethora of controversial experimental results and provides an excellent basis for further computational studies on nanodomains. Furthermore, the results highlight the role of cholesterol as a key player in the modulation of nanodomains for membrane protein function.

## Introduction

Since the introduction of the concept^1^, lipid rafts have been in the limelight of membrane research. The raft model suggests that biological membranes include dynamic nanometre scale functional domains enriched in cholesterol^2^. A considerable body of evidence suggests that nanoscale domains are vital for many key processes such as signal transduction, membrane trafficking, and membrane protein function^2–4^. While the discussion regarding the existence of rafts or the interpretation of related experimental data continues^5, 6^, it is clear that biomembranes are highly heterogeneous, the heterogeneity is crucial for cellular function, and the role of cholesterol in membrane nanodomains is important^4, 7^.

**Figure 1:**
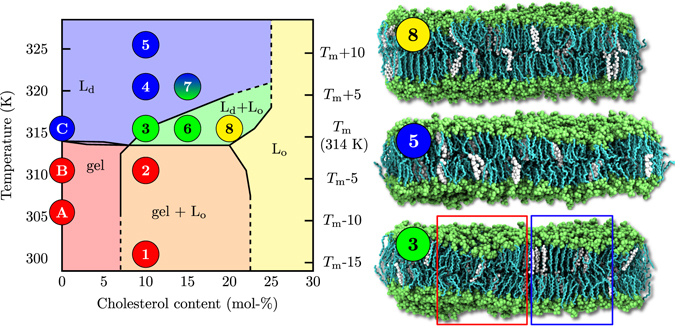
Left: Phase diagram with phase boundary lines as suggested by Vist and Davis^13^. Liquid–liquid coexistence is expected in the L_d_ + L_o_ regime (green). The L_o_, L_d_, gel and gel + L_o_ regions are coloured in yellow, blue, red and orange, respectively. The diagram is shifted upward by 4 K so that the main transition temperature *T*_m_ agrees with that of non-deuterated DPPC. The locations of the simulated systems, also listed in Table S1, are shown by numbers (DPPC–cholesterol) and letters (pure DPPC). Their colours indicate their phases determined from our analyses. Systems 3, 6, and 7 show heterogeneous behaviour. Right: Snapshots of selected systems labelled by the points in the phase diagram: (8) Chol20_316_ (L_o_ phase), (5) Chol10_326_ (L_d_ phase), (3) Chol10_316_ (ordered/disordered/hexagonal), DPPC is shown in cyan and lime (chains and other parts) and cholesterol in white. Water, ions, and lipid chain hydrogens are omitted for clarity. Red and blue boxes highlight disordered and ordered regions, respectively.

How do these biologically relevant nanoscopic domains form and look like in detail? Biomembranes containing thousands of lipid types together with proteins and other macromolecules are too complicated to explore this issue at the level required to understand the roles of individual molecules or their structural features. Therefore, a feasible strategy is to begin with the simplest model membranes that grasp only the most essential aspects of their biological counterparts, and then to move forward step-by-step in complexity. In this work, we employ the simplest model in which nanodomains might form^8, 9^: the well characterized mixture^10, 11^ of cholesterol and dipalmitoylphosphatidylcholine (DPPC). The canonical phase diagram for the DPPC–cholesterol system (see Fig. 1) was first suggested by the theoretical model of Ipsen *et al*.^12^ and a set of experiments by Vist and Davis^13^. Above the main transition temperature of DPPC, this phase diagram states that when added to the DPPC bilayer in the liquid-disordered (L_d_) phase, cholesterol induces a uniform liquid-ordered (L_o_) phase at concentrations above ~20 mol-%. At intermediate cholesterol concentrations of approximately 10–20 mol-% and at temperatures slightly above *T*_m_, the phase diagram by Vist and Davis predicts that the L_o_ phase coexists together with the L_d_ phase. In essence, the Vist–Davis phase diagram suggests that the DPPC–cholesterol system is the minimal model for the consideration of cholesterol-induced lateral membrane heterogeneity. This phase diagram has been discussed thoroughly in recent reviews^8, 14–16^ and idealized models have attempted to explain it (see refs 17–22) yet none of them has achieved the status of a *de facto* model^23, 24^. This is quite surprising given the “simplicity” of the DPPC–cholesterol system and the importance of understanding the physical principles that underlie nanodomain formation. Definitely a valid model consistent with and able to explain the vast amount of experimental data is called for.

The presence of the L_o_/L_d_ phase coexistence in DPPC-cholesterol membranes has been challenged by a picture of a continuous transition between the liquid phases^14^, and a number of dissimilar phase diagrams have been measured^8^ for this mixture since that of Vist and Davis^13^. Considering the large number of studies performed on this mixture, it is puzzling how few of them have actually captured behaviour consistent with the coexistence of L_o_- and L_d_-like components. Only some very accurate techniques, such as NMR and EPR^13, 25, 26^, volumetric measurements^27^, Raman spectroscopy^28^, and quartz crystal microbalance with dissipation monitoring^29^ have detected this phenomenon.

However, unlike many ternary mixtures, the DPPC–cholesterol system does not separate into micrometre-sized phases that can be detected by fluorescence microscopy, implying that the possible sizes of the domains — if they really exist — are in the sub-micrometre scale. This suggests that instead of macroscopic phase separation, the experimental observations could be explained by the formation of ordered nanodomains, *i*.*e*. a membrane would exhibit a single yet heterogeneous phase^30^. We emphasize that we refer to a phase as a macroscopically homogeneous equilibrium state of matter, which is unaffected if it is mechanically isolated from a phase-separated system. Notably, liquid–liquid phase separation of ternary mixtures in liposomes will proceed until only two domains exist, one for each phase. Domains, on the other hand, can be small and transient and arise even in a single phase system, or near a critical demixing point^9, 22^. The formation of nanodomains within a single phase might be favourable over phase separation in case the cost of creating domain boundaries, *i*.*e*. line tension, is sufficiently reduced by, *e*.*g*., linactants or curvature with respect to mixing entropy and other possible contributions disfavouring domain formation^9, 31^.

Experimental studies using FRET^32^ or molecular acoustics and calorimetry^33^ are in line with this view, and the existence of nanodomains at fairly high cholesterol concentrations is also directly supported by recent neutron scattering experiments^34, 35^. Nanodomains have also been observed in DPPC–cholesterol monolayers using X-ray diffraction^36, 37^. It is important to note that many experimental techniques, such as spectroscopic ones, do not provide information on the size and the arrangement of the ordered domains. Therefore it is not clear whether they really detect two phases or whether the two distinguishable signals arise from a heterogeneous single phase that can be described by, *e*.*g*., a modulated phase^20^ or a microemulsion^21^, or whether they arise from critical fluctuations^22^. Indeed, the single-phase nanodomain model is perfectly compatible with all experimental results that have been interpreted as evidence for the presence of liquid–liquid phase coexistence^13, 25–29^.

As to simulations, studies employing atomistic models have not captured lateral heterogeneity in DPPC–cholesterol systems^38, 39^, and recent attempts to probe the phase diagram using united-atom^40^ or coarse-grained models^41, 42^ have not been more successful. If nanoscale domains are the proper realization of the debated coexistence region of the phase diagram of Vist and Davis, then this discrepancy may arise from a number of possible factors. These include limitations in simulation time and size scales, incorrect thermodynamic conditions studied in simulations, or inadequate simulation models. On the other hand, either true L_o_/L_d_ phase coexistence or the formation of nanoscopic domains have been observed in studies using lattice models^12, 43–46^ and molecular dynamics (MD) simulations of very coarse-grained models^35, 47^.

Here, we employ an extensive set of microsecond-scale atomistic MD simulations to probe the phase behaviour of binary DPPC–cholesterol bilayers and the spontaneous formation of nanoscale structures in systems of this mixture. The objective is to demonstrate that a state-of-the-art atomistic model can describe the exceptionally complex DPPC–cholesterol system and its phase behaviour in full agreement with the vast experimental data reported on this system. Furthermore, by focusing on the most exciting region of the phase diagram where nanodomain formation is expected, we aim to shed light in atomistic detail on one of the evergreen problems in membrane biophysics: how does cholesterol give rise to the formation of nanoscale domains, what is their structure, and how this knowledge could be used to better understand the functions of nanodomains in biomembranes.

We show below that the model employed here is consistent with the regions corresponding to homogeneous phases in the phase diagram found by Vist and Davis. Most importantly, we observe — for the first time in atomistic MD simulations — the spontaneous formation of a heterogeneous phase in which cholesterol-rich ordered nanodomains reside in a disordered membrane. The nanodomain structure turns out to be exceptional in the sense that it contains a cholesterol-poor hexagonally packed lipid nanocluster within a fluid cholesterol-rich nanodomain — “nanostructures within nanodomains”. This structure arises in the coexistence region of the Vist–Davis phase diagram^13^ that has traditionally been associated with L_o_/L_d_ phase separation. Within the fluid nanodomains, cholesterol positions itself to its boundary regions, which is expected to promote its availability for cholesterol-binding membrane proteins, translocation, and thereby transmembrane transport of cholesterol. Highlighting the unique nature of cholesterol, we further find that the observed structural reorganization is specific to cholesterol and is not observed when cholesterol structure is slightly altered. We close the article by discussing how the model and the present findings may help to understand the functionality of nanoscale membrane domains and the role of cholesterol in biomembranes.

## Results

### Calibration of the Phase Diagram for the Used Simulation Model

We begin by calibrating the phase diagram in ref. 13 with the *T*_m_ of DPPC in the employed Slipids simulation model^48, 49^. Based on careful comparison of both structural and dynamic features of pure bilayers described in Section S3, we place the *T*_m_ of the simulated DPPC system at ~308 K, a few degrees below the experimental value of 314 K^50^ (note that the phase diagram in ref. 13 is based on deuterated DPPC, whose *T*_m_ is ~310 K^50^).

Based on this information, we label our systems as Chol%_*T*_, where % is the percentage of cholesterol in the system and *T* is the simulation temperature shifted up by 6 K to allow for direct comparison to experiment. The left panel in Fig. 1 highlights the 11 points in phase space that we simulated for varying combinations of temperature and cholesterol concentration (see also Table S1).

### Liquid Nanodomains Are Observed Under Conditions Where Liquid–Liquid  Coexistence is Expected

We proceed to study the behaviour of the bilayers in the eight points marked by numbers in Fig. 1. These points are coloured based on which phases they are in, as described below. As the main criterion for the gel, L_o_, and L_d_ phases we used both the deuterium order parameter for the lipid acyl chains obtained from NMR measurements (see below) and membrane area per lipid and thickness obtained from X-ray scattering (see Sections S4.1 and S4.5). Notably, the agreement with the phase diagram of Vist and Davis^13^ (background colours) is striking in the regions of homogeneous phases (see Section S4 for results on these systems). Additionally, liquid-like nanodomains originate precisely in the region whose heterogeneity was originally associated with phase coexistence^13^, as described below. The phase behaviour found by the simulations and shown in Fig. 1 was confirmed by additional simulations (see Section S1.5).

Snapshots of selected systems are shown on the right in Fig. 1. Chol20_316_ (top panel) and Chol10_326_ (middle panel) systems display the expected homogeneous L_o_ and L_d_ phases, respectively. However, the Chol10_316_ (bottom panel) system contains both ordered and disordered regions. Similar behaviour is also observed in the Chol15_316_ and Chol15_321_ systems (not shown). Interestingly, the spatial heterogeneity in the Chol10_316_ and Chol15_316_ systems is stable, whereas the chain conformations in the Chol15_321_ system are very dynamic. Due to the highly dynamic nature, the features of the Chol15_321_ system are not sufficiently captured with the time-averaged analyses employed here. The discussion in the main article will therefore focus on the analyses on the more stable nanodomain observed in the Chol10_316_ system, while the results for the other heterogeneous systems are shown in the SI and discussed below only briefly. The presence of the heterogeneities is validated by spatially and temporally resolved analyses that also confirm the homogeneous nature of the other studied systems.

**Figure 2:**
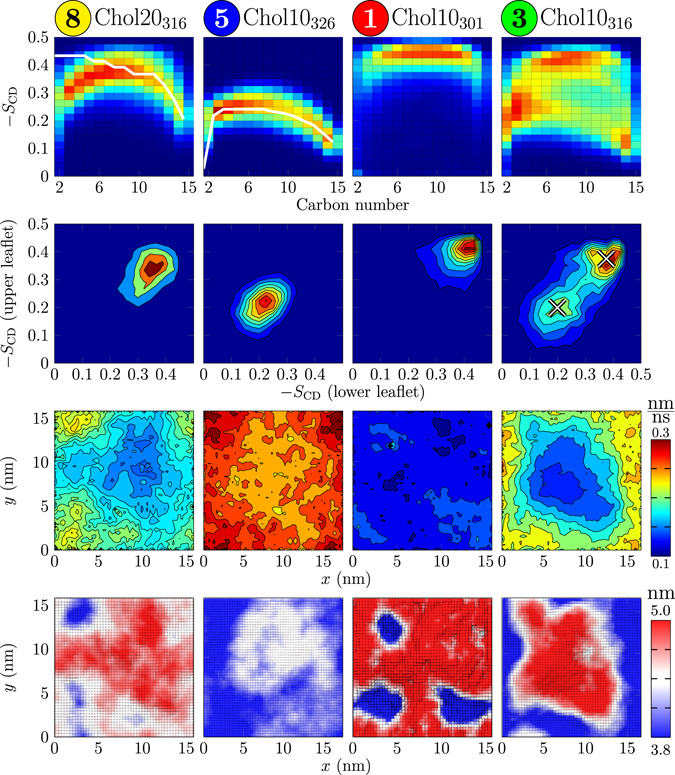
Results shown for systems labelled by the points in the phase diagram (Fig. 1): (8) Chol20_316_ (L_o_ phase), (5) Chol10_326_ (L_d_ phase), (1) Chol10_301_ (gel), (3) Chol10_316_ (ordered/disordered/hexagonal). First row: Deuterium order parameter distributions along the *sn*-2 chain of DPPC. Available experimental data are shown in white (full line): For the Chol20_316_ system (8), given here are the data measured for the L_o_ phase (DPPC + 40 mol-% cholesterol at 308 K)^51^. For the Chol10_326_ system (5), the experimental data are for the L_d_ phase system (pure DPPC at 314 K)^52^. Second row: The spatial correlation of lipid chain order (*sn*-2 chain of DPPC) between the leaflets. Third row: In-plane lipid displacement map. Fourth row: Thickness maps.

The deuterium order parameter distributions along the *sn*-2 chain of DPPC are shown for selected systems on the top row of Fig. 2 (for data on all systems, see Section S4.6). The Chol20_316_ (leftmost panel) and Chol10_326_ (2nd panel from the left) systems show relatively narrow distributions, in line with the behaviour of a homogeneous phase, and their average values are in excellent agreement with experimental data on L_o_^51^ and L_d_^52^ phases, shown with white lines. For the former, the disagreement in the beginning of the acyl chain is caused by limitations when applying and interpreting NMR on fully deuterated lipids^53, 54^. The shape of the profiles measured using specifically deuterated DMPC in mixtures with cholesterol is in agreement with our profiles^55^. The gel phase Chol10_301_ system (third panel from the left) also shows a narrow distribution, however experimental data are not available for comparison. Other single-phase systems follow the same trends, with Chol10_305_ and Chol10_321_ systems showing slightly less pronounced gel and L_d_ behaviour (see Section S4.6).

The order parameter distribution of the Chol10_316_ system (rightmost panel on the top row in Fig. 2) is significantly broader than the others and contains contributions from all homogeneous single-phase systems. Indeed, it can be reproduced as a linear combination of the profiles of the homogeneous phase systems (three leftmost panels). According to a least squares fit, the Chol10_316_ system consists of 37% disordered L_d_-like, 42% ordered L_o_-like, and 21% gel-like (see below) DPPC chains, consistent with the heterogeneity detected also by experiments^13, 25–29^. While this linear combination reproduces the original profile within an error of ~10%, it is still only suggestive: the gel-like and L_o_-like systems employed in the fitting have 10% and 20% cholesterol, respectively, while the local concentrations in the related regions in the heterogeneous systems likely deviate from those. Notably, as we argue in the discussion, this heterogeneous behaviour should not be interpreted as multiple coexisting phases, but rather as a heterogeneous phase with possible resemblance to a microemulsion.

The order parameter distribution of the Chol15_316_ system (see Section S4.6) can be reproduced as a combination of 25% disordered, 73% ordered, and 2% gel-like components, indicating that an increase in cholesterol content promotes the ordered-like structure while the gel-like contribution decreases. The Chol15_321_ system (see Section S4.6) cannot be reproduced by such a linear combination as it falls between that of ordered and disordered behaviour, indicating dynamic coexistence in which lipids constantly change between these two states.

Analysis of order parameter correlation between leaflets also reveals the presence of heterogeneities, see the second row in Fig. 2 for data of selected systems. This analysis further supports the presence of disordered, ordered, and gel-like components in the Chol10_316_ system, and agrees qualitatively with the fractions of each component estimated from the fits to the distributions on the top row in Fig. 2. The probability density located on the diagonal suggests that the locations of ordered and disordered regions are correlated across the membrane, *i*.*e*. the systems display membrane registry. Similar effects were also observed for the Chol15_316_ system, while the Chol15_321_ system again shows dynamic intermediate behaviour. Localized distributions characteristic to a homogeneous phase are observed for the other systems (see Fig. S6).

We next examine how the regions of different lipid chain order are spatially located using membrane thickness maps, shown on the bottom row in Fig. 2. While other systems show fairly uniform thickness that agrees well with experimental estimations (see Section S4.5) with the exception of the thin regions in the Chol10_301_ system due to lipid ripples, the shape of the thicker nanodomain in Chol10_316_ system is clearly visible. Importantly, contrary to previous works, this ordered domain self-assembles spontaneously in our simulation. The thickest regions (in red) correspond to the gel-like component observed earlier. Its presence is also evidenced by the map of average in-plane lipid displacements (“jump map”) calculated over 1 ns intervals, shown on the third row of Fig. 2. Clearly, the gel-like core is rather immobile. Still, the thickness of the immobile region is less than that of the DPPC gel phase (see discussion in Section S4.5), and the gel-like contribution (Chol10_301_ system, 3rd column) seems to be absent in the order parameter distributions (top row) and order parameter correlation plots (2nd row) for the Chol10_316_ system (4th column) in Fig. 2. These observations suggest that the gel-like region might be a natural part of the structure of the liquid nanodomain. This is supported by a recent study on ternary lipid mixtures that observed similar hexagonally-packed and cholesterol-depleted regions in the L_o_ phase^56^. The core region is surrounded by a more mobile annular region rich in cholesterol. These two regions constitute the ordered nanodomain that is surrounded by disordered regions. Furthermore, the gel-like core might possibly act as a nucleation centre for nanodomain formation, especially in systems near *T*_m_, where gel-like packing might transiently arise due to critical fluctuations. However, this seems unlikely as heterogeneity is also observed in the Chol15_316_ system, where no gel-like component is present (see Section S4.7). This system seems to instead contain disordered nanodomains in an ordered membrane, similar to those observed for DPPC–cholesterol monolayers^57^ (see Section S4.5). Alternatively, the incomplete formation of an ordered nanodomain in this system might be limited by the simulation box size. Furthermore, the dynamic Chol15_321_ system does not show the formation of a well shaped domain. Other systems show homogeneous behaviour in terms of both thickness, order, and mobility (see data for all systems in Sections S4.5, S4.6 and S4.7).

Concluding, our simulation model is in excellent agreement with experimental data and reproduces the phase diagram within the regions associated with homogeneous phases. However, in the proposed L_o_/L_d_ phase coexistence region, instead of macroscopic phase separation we observe the formation of a liquid heterogeneous phase formed by ordered nanodomains in an otherwise disordered bilayer. Depending on the temperature, these domains are either stable or dynamic within the simulation time scale.

### Cholesterol is Excluded from the Most Ordered Regions

Next, we set to study the structure of the nanodomains in detail. In their recent study on phase-separated ternary lipid mixtures^56^, Sodt *et al*. noticed that cholesterol preferentially partitions into the boundary regions of the L_o_ phase, and the ordered nanodomain in the Chol10_316_ system indeed shows similar behaviour.

**Figure 3:**
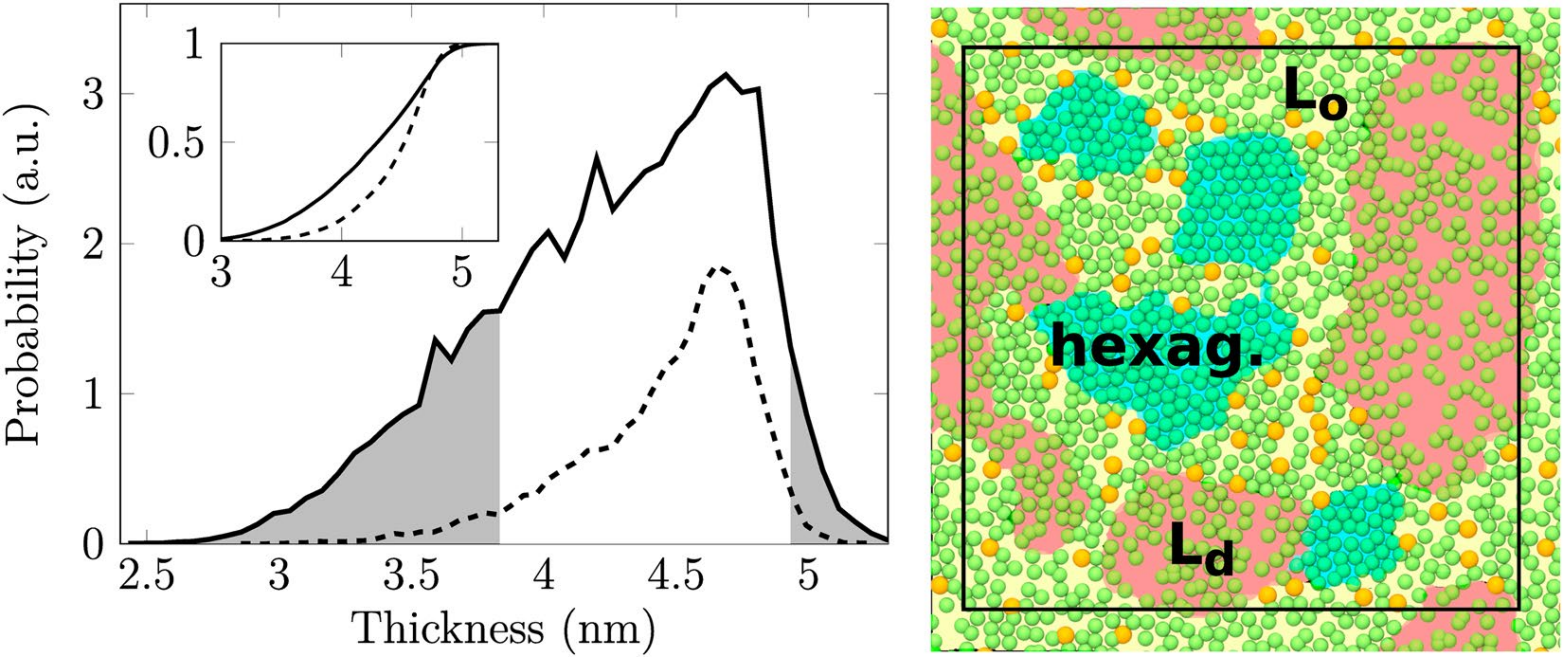
Left: Thickness histogram for the Chol10_316_ system. The inset shows the normalized cumulative distributions. Solid lines show data collected from the whole system and dashed lines data collected from locations occupied by cholesterol. Grey fill highlights regions with a small local concentration of cholesterol. Right: The proposed domain structure; DPPC chains are shown in green and cholesterol in orange. The estimated locations of lipids displaying L_o_-like, L_d_-like, and hexagonally packed regions are shaded with yellow, red, and blue, respectively. The simulation box is drawn in black.

The histogram of local membrane thicknesses, shown on the left in Fig. 3 (see also Section S4.10) shows that cholesterol locates to a significant extent in thicker ordered regions, however cholesterol is excluded from the very thickest regions of the membrane. The neighbour distributions of lipid chains (Section S4.11) show that some lipid chains in the Chol10_316_ system have six nearest neighbours, indicating hexagonal packing. Repeating this analysis considering cholesterol as a lipid chain reveals that cholesterol does not substantially perturb the first coordination shell of DPPC chains. We conclude that the thickest and least mobile regions in the Chol10_316_ system are hexagonally packed and poor in cholesterol, and reside in the core of the domain. It is this core region that gives rise to the gel-like order parameters observed in the order parameter distribution for this system (see top row in Fig. 2 All these observations are in agreement with the packing of the ordered phase reported for the ternary (DPPC–DOPC–cholesterol) system^56^.

The present observations are summarized on the right in Fig. 3 which shows the typical arrangement of lipid chains and cholesterols together with the estimated locations of the ordered domain, disordered bulk regions, and the hexagonally packed core in the Chol10_316_ system. The specific positioning of cholesterol in the structurally perturbed interface region induces cholesterol flip–flops (see Section 4.12). Further results on the positioning of cholesterol are presented in Sections S4.10 and S4.11.

Concluding, our results highlight the fine structure of the nanodomain with a hexagonally packed core and a cholesterol-rich boundary region.

### Cholesterol Asymmetry Affects the Domain Stability

In its steroid backbone, cholesterol has two distinctly different faces: the alpha-face that is smooth, and the beta-face that is rough. The importance of the two unlike faces in the formation of liquid–liquid phase separation in ternary lipid membranes was recently highlighted by the work of Sodt *et al*.^56^, who noticed that the L_o_ phase is favored by the smooth face, leaving the rough side to face the L_d_ phase. We do not find such orientational preference of cholesterols at the domain boundary in our binary system (data not shown).

Despite this, we study the importance of cholesterol asymmetry by replacing cholesterols in the Chol10_316_ system by their demethylated analogue (18,19-di-nor-cholesterol, DMchol), *i*.*e*. cholesterol with two smooth faces (see Section S1.3). This modification is performed once the domain has formed, and the structure of the domain is monitored during further simulation. We observe that during a period of 700 ns this change from cholesterol to DMchol results in a shrinking of the ordered domain to approximately half of its original size. The average area per lipid increases slightly, suggesting that the smooth analogue has a weaker condensing effect than cholesterol, in agreement with previous computational and experimental studies^58–60^. The lack of the rough face takes the system to a disordered membrane with a small hexagonally packed domain that shrinks in time, and at long times this domain will clearly eventually disintegrate (see Section S4.13).

Concluding, the asymmetric structure of cholesterol might be important for the formation of nanodomains. However, more systematic studies on the effect of cholesterol analogues need to be performed to further clarify this issue.

## Discussion

In this work, we simulated large DPPC–cholesterol bilayers and probed their phase behaviour through microsecond-scale atomistic resolution simulations. Based on exhaustive analyses, our system is consistent with the behaviour of the homogeneous phase regions summarized in the canonical phase diagram proposed by Vist and Davis^13^. However, in the coexistence region of this phase diagram we observe for the first time — instead of phase separation — the spontaneous formation of a single heterogeneous phase in which ordered cholesterol-rich nanodomains reside among an otherwise disordered and cholesterol-poor membrane. This behaviour cannot be explained by two coexisting phases since the domain does not behave like a phase: simulations using a system with the composition of the domain only (corresponding to system 8 in Fig. 1) show uniform L_o_-like behaviour. Taken away from a phase-separated system, a proper phase should not change its behaviour. Based on our results, inter-leaflet coupling between ordered domains is evident. In fact, lipid chain ordering is strongly spatially correlated between the leaflets (second row in Fig. 2).

The ordered nanodomains observed in the Chol10_316_ system show an irregular shape with a diameter of approximately 10 nm. Due to the slow reshaping of the domain we are, however, unable to predict whether it will eventually be more round. Still, this size of the nanodomain fits the suggested picture of ordered lipid nanodomains^30, 32^. Importantly, most of the experimental evidence supporting liquid–liquid phase coexistence in this system is obtained indirectly and can be explained by the existence of small ordered domains, similar to those observed in our work.

The nanodomains we observed are quite exceptional since they have complex internal structure: they contain a cholesterol-free hexagonally packed lipid nanocluster core surrounded by a fluid cholesterol-rich nanodomain. That is, the nanodomains we observed are actually nanostructures within nanodomains, where cholesterol partitions around the hexagonally packed core, at the interfacial cholesterol-rich ordered region between the core and the disordered membrane phase. This, together with the recent observations suggesting that cholesterol partitions into phase boundaries with a preferential orientation in a ternary lipid system^56^, led us to study the preference of cholesterol faces for the ordered and disordered regions. Surprisingly, no such preference was found in our binary mixtures. This suggests that the orientation of cholesterol at domain boundaries might result from differences in the interactions of the cholesterol faces with saturated and unsaturated lipid chains rather than from their preferences for ordered or disordered configurations of lipid chains^61^.

The smoothening of cholesterol in our Chol10_316_ system — where we observed a well-shaped nanodomain — resulted in the disappearance of the ordered component and the shrinkage or disappearance of the gel-like core. Furthermore, cholesterol can induce 2D ordering, while DMchol favours linear arrangements^59^. This difference might explain why the domain-forming capabilities of cholesterol and DMchol are different despite having similar local ordering abilities. Though a synthesis route for DMchol was very recently discovered^62^, its effects on membrane phase behaviour have not been studied experimentally. However, studies on lanosterol — a cholesterol analogue with two rough faces— suggested that it has a weaker ordering effect compared to cholesterol^63^ and that the liquid–liquid coexistence region was not present in lipid–lanosterol mixtures^64^, in agreement with our results, further supporting the important role of cholesterol asymmetry.

The ordered nanodomains consist of cholesterol-free hexagonally packed core regions surrounded by an ordered and liquid cholesterol-rich interface region around the core. The hexagonal packing within the nanodomains is in agreement with very recent neutron scattering experiments^34^. The suggested structure also closely resembles that determined for a ternary DOPC–DPPC–cholesterol membrane^56^. The thickness of the hexagonally packed regions is less than that measured for gel phase DPPC. This difference cannot be explained by tilting as no collective tilting of lipid chains in the hexagonally packed regions was observed. Therefore, we do not consider the hexagonally packed regions to be in a true gel state. This view is further supported by a comparison of the results for the Chol10_301_ and Chol10_316_ systems in Fig. 2.

Altogether, we have shown that a modern all-atom simulation model is able to reproduce and explain the experimentally resolved behaviour of the complex mixture of DPPC and cholesterol, a task in which previous atomistic and coarse-grained models have come short (see Section S5). The model captures the debated liquid–liquid heterogeneity through the formation of ordered liquid nanodomains whose formation is driven by cholesterol. The simulation data also revealed that cholesterol selectively organizes itself to the boundary of the nanodomain, where it is better available for cholesterol-binding proteins, and where it carries ouf flip–flops more frequently than elsewhere in the membrane, thus suggesting that nanodomain boundaries promote transmembrane transport of cholesterol and thus contribute to the asymmetric cholesterol distribution.

The simulation findings are consistent with the indirect observations of coexisting liquid phases^13, 25–29^ as well as the new data^32–35^ suggesting the existence of ordered domains within a disordered lipid sea. All these data together with ours, and the lack of direct evidence for phase coexistence — most importantly that of fluorescence microscopy — suggest that the phase diagram of Vist and Davis^13^ requires a revision in the L_o_/L_d_ coexistence region. The nature of this heterogeneous region which — instead of phase coexistence — might be described by a single phase in the form of, *e*.*g*., a modulated phase, a microemulsion, or critical fluctuations is unclear and under ongoing debate^9, 20–22, 47^. The domain substructure suggests that — if the system is really a microemulsion — the domains might be stabilized by the cholesterol-rich region of the nanodomain acting as a linactant instead or in addition to, *e*.*g*., membrane curvature^21^. Actually, we observed that the line tension associated with the domain boundary is much smaller in this binary lipid mixture as compared to a ternary one, see Sections S2.6 and S4.9. This would partially explain why the binary mixture does not undergo macroscopic phase separation. However, due to the used method’s inability to account for the complexity of the nanodomain substructure, we take these results with a grain of salt. Therefore, the physical models describing the observed phenomena, as well as the large-scale structural and dynamic behaviour of systems with multiple nanodomains remain to be discussed in future studies. In this context — given the consistency of the present simulation results with the vast amount of experimental data on the DPPC–cholesterol system — the model described in this work provides indeed a very promising basis to explore the physical principles that control nanodomain formation, the lifetimes of the nanodomains, and the structure and dynamics of nanodomains interacting with and hosting membrane proteins.

Are the present results biologically relevant? DPPC is quite uncommon in the human body except for the pulmonary surfactant, where it accounts for approximately half of all phosphatidylcholine lipids, which in turn represent the dominant phospholipid type^65^. DPPC is considered to be responsible for the surface tension lowering capability of the surfactant^66^. Notably, the concentration of cholesterol in the pulmonary surfactant is also smaller than in most of the membranes, ranging around 10 mol-%^65^, *i*.*e*. within the range considered in this study. Hence, although the pulmonary surfactant is complex and contains also unsaturated lipids, phospholipids from various other classes, as well as surfactant proteins^66^, the binary mixture of DPPC and cholesterol is nevertheless a good approximation of the pulmonary surfactant with the capability to undergo domain formation. In pulmonary surfactant membranes, the formation of cholesterol-rich and cholesterol-poor domains^67^ is crucial, since the partitioning of surfactant-associated proteins to such domain structures plays a role in their activation^66^. In plasma membranes rich in cholesterol, the function of rafts has been discussed to be associated with interactions of cholesterol with saturated lipids, hence the present conclusions for cholesterol and saturated DPPC are expected to hold for quite a few mixtures of cholesterol with saturated lipids. More importantly, our results predict that in biomembranes with functional nanoscale domains, cholesterol has several potential functions. First, cholesterol may bind specifically with membrane proteins and thereby modulate their conformation and function, or cholesterol may modulate membrane physical properties to match the conditions that promote the activation of a given membrane protein. Related experimental^68, 69^ and simulation^70–72^ data are consistent with this view. Also, as discussed by Sodt *et al*.^56^, the availability of cholesterol at the boundaries of small domains might promote biological function, rendering cholesterol molecules more accessible to proteins associated with these boundaries. The dependence of nanodomain formation on the structural asymmetry of the cholesterol ring may explain, in part, why nature prefers cholesterol over other sterols in eukaryotic membranes. Furthermore, in order to activate themselves, membrane proteins should be able to organize and maintain their conformational states for sufficiently long times, including slow events such as the binding of key lipids to their specific lipid binding sites in the protein, and the binding of several proteins undergoing dynamic conformational changes in oligomer formation. This implies that the lifetime of the domain hosting the proteins should be long enough.

## Methods

Large DPPC bilayers (~1000 lipids) with various amounts of cholesterol were simulated at various temperatures representing multiple points in the phase diagram (see Fig. 1 and Table S1). The bilayers were modelled using the Slipids force field^48, 49^ and simulated until their area had equilibrated (up to 1.3 μs) after which 100 ns of data were collected for analyses. For a thorough description on the construction, simulation and analysis of the systems, see Sections S1 and S2. The simulation data and all the files required to reproduce them are available at doi:10.5281/zenodo.439066 and doi: 10.5281/zenodo.439080.

## Electronic supplementary material

Supporting Information for Nanoscale Membrane Domain Formation Driven by Cholesterol
