# Supplementary material for: Nanoscale Membrane Domain Formation Driven by Cholesterol
Source: Sci Rep. 2017 Apr 25;7:1143. doi: 10.1038/s41598-017-01247-9 (PMC5430823; doi:10.1038/s41598-017-01247-9)
Supplement: Supplementary file 1 — Supporting Information for Nanoscale Membrane Domain Formation Driven by Cholesterol [file 41598_2017_1247_MOESM1_ESM.pdf]

Supporting Information for
Nanoscale Membrane Domain Formation Driven by Cholesterol

Matti Javanainen, Hector Martinez-Seara, Ilpo Vattulainen
hseara@gmail.com,ilpo.vattulainen@helsinki.fi

Contents

S1 Simulation Methods **2**
S1.1 List of Simulated Systems . . . 2
S1.2 Construction of Simulated Systems . . . 2
S1.3 Parametrization of Demethylated Cholesterol . . . 2
S1.4 Simulation Parameters . . . 3
S1.5 Additional Simulations . . . 3
S1.5.1 Gel-Phase Membrane With Anisoropic Pressure Coupling . . . 3
S1.5.2 Bilayer Self-Assembly and Subsequent Domain Formation . . . 3
S1.5.3 Effect of Lennard-Jones Cutoff . . . 3

S2 Analysis Methods **4**
S2.1 Diffusion Coefficients . . . 4
S2.2 Order Parameter Distribution . . . 4
S2.3 Order Parameter Correlation Between Leaflets . . . 4
S2.4 Lipid Displacement Maps . . . 4
S2.5 Thickness and Thickness Gradient Distributions . . . 5
S2.6 Line Tension Calculation . . . 5

S3 Main Transition Temperature of DPPC **6**
S3.1 Visual Observation . . . 6
S3.2 Area Per Lipid . . . 6
S3.3 Chain Order Parameters . . . 8
S3.4 Lateral Diffusion . . . 8
S3.5 Conclusion . . . 8

S4 Additional Results on DPPC–cholesterol Systems **8**
S4.1 Area Per Lipid . . . 8
S4.2 Radial Distribution Functions . . . 8
S4.3 Mean Squared Displacement . . . 10
S4.4 Lipid Tilt Angle . . . 10
S4.5 Thickness Maps . . . 10
S4.6 Deuterium Order Parameter Distributions . . . 12
S4.7 Average Displacement Maps . . . 12
S4.8 Order Parameter Correlation between Leaflets . . . 12
S4.9 Line Tension Calculation . . . 13
S4.10 Thickness Distributions . . . 17
S4.11 Nearest Neighbor Distributions . . . 17
S4.12 Location of Cholesterol Flip-flops . . . 17
S4.13 Effects of Cholesterol Ring Demethylation . . . 18

S5 Comparison with Studies Employing Other MD Models **20**

This Supporting Information contains additional information on the construction of the simulated systems, additional simulations performed to verify the conclusions presented in the main paper, and descriptions of the employed analysis methods. Also, some relevant results left out from the main paper are presented here.

S1 Simulation Methods

S1.1 List of Simulated Systems

The systems simulated to probe the phase diagram are listed in Table S1. Simulations A, B, and C were employed to resolve the main transition temperature of pure DPPC (see Section S3).

Table S1: Studied systems with numbering referring to those shown in the phase diagram in the main text. For information on additional simulations excluded from the main text, see Section S1.5. Here T_{eff} stands for the temperature scaled to match the phase diagram of Vist and Davis [1], whereas T_{sim} is the real simulation temperature (see Section S3). Simulation time is denoted as t, and APL stands for the average area per (phospho)lipid (see Section S4.1). The end states denote which kind of lipid behavior is observed in the systems based on multiple results presented in the main text and this document. "D/O" refers to heterogeneity with characteristic features of the liquid-disordered and liquid-ordered phases, while it is not true $\mathrm{L_o}$/$\mathrm{L_d}$ phase coexistence. The abbreviation "hexag." refers to hexagonal packing of lipid chains (see main text and below), and "Dyn" refers to dynamic heterogeneity. DPPC and CHOL stand for the number of DPPC and cholesterol molecules, in respective order.

No.	Name	T_{eff} (K)	T_{sim} (K)	DPPC	CHOL	t (ns)	APL (Å^2)	End state
A	$\mathrm{Chol0_{300}}$	306	300	1152	0 (0%)	500	51.9±0.5	Gel
B	$\mathrm{Chol0_{305}}$	311	305	1152	0 (0%)	500	53.4±0.6	Gel
C	$\mathrm{Chol0_{310}}$	316	310	1152	0 (0%)	300	59.8±0.4	$\mathrm{L_d}$
1	$\mathrm{Chol10_{301}}$	301	295	1032	120 (10.4%)	680	49.8±0.2	Gel
2	$\mathrm{Chol10_{311}}$	311	305	1032	120 (10.4%)	1000	52.4±0.3	Gel
3	$\mathrm{Chol10_{316}}$	316	310	1032	120 (10.4%)	1400	56.2±0.3	D/O/hexag.
4	$\mathrm{Chol10_{321}}$	321	315	1032	120 (10.4%)	500	59.5±0.2	$\mathrm{L_d}$
5	$\mathrm{Chol10_{326}}$	326	320	1032	120 (10.4%)	500	61.2±0.4	$\mathrm{L_d}$
6	$\mathrm{Chol15_{316}}$	316	310	976	176 (15.3%)	1000	56.8±0.5	D/O
7	$\mathrm{Chol15_{321}}$	321	315	976	176 (15.3%)	1000	60.1±0.4	Dyn. D/O
8	$\mathrm{Chol20_{316}}$	316	310	912	240 (20.8%)	500	56.8±0.3	$\mathrm{L_o}$

S1.2 Construction of Simulated Systems

Initially DPPC–cholesterol systems with a total of 288 lipids were constructed. These systems contained 0, 30, 44, and 60 cholesterol molecules corresponding to 0, ∼10, ∼15, and ∼20 mol%, respectively. The systems were simulated for 200 ns at 310 K (simulation temperature) with the simulation parameters identical to those employed in the larger production simulations described in Section S1.4. After this equilibration of the small patches, the system coordinates were multiplied by 2 in both directions along the membrane plane to create a system with a total of 1152 lipids including either 0 (0 mol%), 120 (10 mol%), 176 (15 mol%), or 240 (20 mol%) cholesterol molecules. These large patches were employed as initial structures for all production simulations and only the target temperature of the thermostat was modified.

S1.3 Parametrization of Demethylated Cholesterol

The cholesterol with a demethylated ring (DMchol) was constructed by replacing the two out-of-plane methyl groups by hydrogens similarly as in Ref. 2. The charges of these replacement hydrogens were set to match the charges on similar hydrogens in the cholesterol structure. The leftover charge from the methyl group was placed on the carbon to which the replaced methyl groups used to be connected. The partial charges in the

hydrophobic ring structure are small and therefore the charge fitting procedure was omitted. Whenever the angle or dihedral parameters were missing for an interaction involving a replacement hydrogen, the old values involving the removed methyl carbon were employed instead.

S1.4 Simulation Parameters

The simulations were performed with a time step of 2 fs and the simulation length varied between 300 ns to 1.4 µs. Lipid reorganization associated with phase transitions can be slow and the durations of the simulations were therefore chosen based on equilibration of the area per lipid profiles. After the convergence of these profiles was observed, a further 100 ns of data was collected for each system for the analyses. This data was saved every 100 ps. The Slipids force field was employed for the lipids [3] and cholesterol [4], while the TIP3P model [5] was used for water. A simple cut-off scheme with a cut-off length of 1 nm was employed for Lennard-Jones interactions. The same cut-off was used for the real part of the calculation of the electrostatic interactions beyond which the PME scheme [6] was employed. The neighbor list with a radius of 1 nm was updated every 10 steps. Dispersion correction was applied to both energy and pressure. Temperature of the membrane and solvent were coupled separately with the velocity rescaling thermostat [7] with a relaxation time constant of 0.5 ps. The pressure was maintained at 1 bar with a semi-isotropical Parrinello–Rahman barostat [8] with a relaxation time constant of 1 ps. All simulations were run with the version 4.6.X of the GROMACS package [9].

S1.5 Additional Simulations

In addition to the systems listed in Table S1, we also considered some additional test scenarios.

S1.5.1 Gel-Phase Membrane With Anisoropic Pressure Coupling

Since the gel-phase system at 301 K (295 K simulation temperature) was unable to completely relax to the tilted chain conformation, we employed an anisotropic barostat which allowed the two lateral membrane dimensions to vary independently of each other. Our aim was to remove the packing restraints imposed by the requirement of a square simulation box in the membrane plane. However, the observed chain interdigitation still took place and no qualitative differences between the trajectories obtained with isotropic and anisotropic coupling schemes were observed.

S1.5.2 Bilayer Self-Assembly and Subsequent Domain Formation

We also verified that the observed heterogeneous behavior was indeed reproducible in further simulations. To this end, we simulated the formation of the small (288 lipids) DPPC–cholesterol bilayer and the subsequent domain formation. We began by heating the existing bilayer with 10 mol% cholesterol until its structure was destroyed and the independent lipid molecules were randomly distributed in water. The system was then cooled to 316 K (310 K simulation temperature). We observed the bilayer formation in $\sim$320 ns, and the subsequent initial separation of the bilayer into cholesterol-poor and cholesterol-rich regions during the following $\sim$150 ns (data not shown). This observation further confirms the reproducibility of the observed heterogeneous behavior.

S1.5.3 Effect of Lennard-Jones Cutoff

We also ensured that the simulation parameters did not have substantial effects on the results. The Slipids force field was originally derived with Lennard-Jones interactions cut-off at 1.4 nm. However, unofficial tests of the authors have shown that a cut-off of 1 nm (employed in this study) leads to correct bilayer behavior. To verify this conclusion, we also simulated the small patch (288 lipids) with 10 mol% cholesterol at 316 K (310 K simulation temperature), *i.e.*, the system with heterogeneity in the form of a nanodomain, with a Lennard-Jones cut-off of 1.4 nm and observed no qualitative differences (data not shown).

S2 Analysis Methods

Density profiles, diffusion coefficients, and lateral radial distribution functions were directly obtained with the standard tools included in the GROMACS distribution. Areas per lipid and line tensions (see below) were calculated from the output of the GROMACS tool `g_energy`. The neighbor distributions were analyzed laterally based on the method of Mazur [10]. Other analysis methods are explained in the following subsections.

S2.1 Diffusion Coefficients

The lateral mean squared displacements (MSDs) were calculated for the lipids with the GROMACS tool `g_msd`. The center of mass motion of the bilayer was removed in the analysis. In all analyses we only consider the last 100 ns of the trajectories in order to discard the data representing the possible reorganizing of lipids into a domain. Therefore we discuss the qualitative trends on the MSD plots rather than the diffusion coefficient values which would require the analyzed time scale to be an order of magnitude longer for proper normal diffusion regime to appear [11].

S2.2 Order Parameter Distribution

Whereas an averaged order parameter profile describes the average behavior of the bilayer and is meaningful for systems in a uniform phase, the profiles for individual lipids are able to reveal the presence of more and less ordered lipid chain configurations. Therefore, we extracted the distributions of the order parameter profiles along the lipid chain. We employed the *sn*-2 chain of DPPC in the analysis and calculated the average deuterium order parameter profiles separately for each lipid. The deuterium order parameters were calculated separately for each methyl group from the angle between the C–H vector and the z axis from

$$-S_{\mathrm{CD}} = -\left\langle \frac{3\cos^2\theta - 1}{2} \right\rangle . \tag{1}$$

These averaged profiles over each lipid molecule were then binned in a two-dimensional histogram with one axis representing the carbon number along the DPPC chain and the other axis the $-S_{\mathrm{CD}}$ value.

S2.3 Order Parameter Correlation Between Leaflets

The correlation of the lipid chain order, defined by the deuterium order parameter, between the two membrane leaflets provides information on bilayer registry, *i.e.*, on whether locations of the ordered domains in the two leaflets are coupled. The order parameter values were resolved spatially for both bilayer leaflets with the `g_lomepro` tool [12]. A grid spacing of 0.5 nm was employed along the bilayer plane. The average value of the *sn*-2 chain of DPPC was calculated at every grid point separately for both leaflets. These pairs of values were then binned in a two-dimensional histogram based on the average order parameter values. The analyzed trajectory was split into pieces each of which was 10 ns long and data calculated for these shorter pieces were considered as individual samples and therefore contribute independently to the 2-dimensional histogram.

S2.4 Lipid Displacement Maps

Lipid diffusivity is another good indicator of the phase of the studied membrane. Whereas the diffusion coefficients measured for the liquid-disordered phase are slightly higher than those of the liquid-ordered phase, the values for the gel phase are orders of magnitude smaller. As the diffusion coefficients are usually calculated from long-time mean squared displacement data, they do not provide details on spatial domain structure due to lipid exchange between the domains. In contrast, the average displacements over a fixed time interval, acting as a qualitative measure of diffusion, can be spatially separated. Therefore the average lateral displacements of DPPC during 1 ns intervals were analyzed locally. The motion of the center of mass of each lipid was considered. The obtained values were binned in a two-dimensional grid based on the location of the starting points of the displacement vectors in order to spatially resolve areas of higher and lower mobilities.

S2.5 Thickness and Thickness Gradient Distributions

In order to resolve the partitioning of cholesterol between the ordered and disordered domains, we calculated the distributions of the local membrane thickness considering first the whole bilayer and then only the locations occupied by cholesterol molecules. The thickness of the membrane was resolved locally from the interleaflet phosphate–phosphate distance using the `g_lomepro` tool [12] and a lateral grid spacing of 1 nm. These values were binned into a histogram. The thicknesses at the positions occupied by cholesterol molecules were binned into another histogram. These were obtained from the thickness data by linear interpolation at the cholesterol positions in Matlab [13] with the `griddata` function. In order to improve both the statistics and the accuracy of the cholesterol positions, the trajectory was divided into pieces of 1 ns that were analyzed separately. Similarly, the cholesterol positions were recorded at the corresponding times at 1 ns intervals.

In addition to the thickness values, similar analysis was performed on the norms of the thickness gradient, *i.e.* the local rate of change of membrane thickness. The gradients were calculated from the thickness map with the `gradient` function in Matlab [13] and the data was again linearly interpolated at cholesterol positions.

S2.6 Line Tension Calculation

Whether membrane heterogeneity is manifested as small domains or macroscopically separating phases is largely determined by the energetic penalty of forming a domain boundary, *i.e.*, line tension [14]. The effect of this line tension, arising from thickness mismatch, is opposed by entropy and possibly other factors which are currently under debate [14].

Since binary mixtures of DPPC (and other saturated PC lipids) with cholesterol do not display macroscopic (L_o/L_d) phase separation in experiments, the line tension associated with the domains is expected to be small. In addition to the DPPC–cholesterol system, the macrosopically phase separating ternary DPPC–dioleylphosphatidylcholine (DOPC)–cholesterol membrane is analyzed as a control.

We followed the setup of Jiang et al. [15]. We simulated systems containing an artificially constructed boundary between ordered and disordered domains. We considered both a macroscopically phase-separating ternary mixture [16] at 293 K (simulation temperature) and the binary mixture showing nanoscopic domain formation at 310 K (simulation temperature, effective temperature of 316 K). The final structures of these systems are shown in Fig. S7. The initial structure of the ternary mixture contained a disordered patch formed of 166 (∼39 mol%) dioleoylphosphatidylcholine (DOPC) molecules, and an ordered patch containing a mixture of 204 (∼48 mol%) DPPC molecules and 52 (∼12 mol%) cholesterols. The binary system consisted of a similar ordered domain with 204 DPPC and 52 cholesterol molecules and a disordered side consisting of 188 DPPC molecules. Therefore, before lateral mixing, the ordered domain contained 20 mol% cholesterol in both binary and ternary systems. The two halves were equilibrated separately and merged thereafter. The number of molecules in the disordered patch was adjusted so that its size matched that of the ordered one. In addition, we also calculated the line tension for a binary system in which cholesterol molecules were replaced by DMchol.

These systems were simulated for 600 ns. The domain boundaries were allowed to stabilize during the first 300 ns after which data were collected for the analyses. This fairly long equilibration time also allowed the cholesterol concentration to equilibrate from the idealized initial structure. The simulation parameters follow those used for the equilibrium simulations except for the pressure coupling scheme, which was adapted from the work of Jiang et al. [15] in order to extract the line tension from the pressure components. The phase boundary was aligned along the z axis, and the simulation box edge was kept constant in the z dimension. In this setup, the line tension Λ associated with the domain boundary is obtained from

$$\Lambda = \frac{1}{2} \left\langle L_x L_y \left[\frac{1}{2} \left(P_{xx} + P_{yy} \right) - P_{zz} \right] \right\rangle , \qquad (2)$$

where P_{ii} are the diagonal components of the pressure tensor and L_x and L_y are the lengths of the simulation box edges that are perpendicular and parallel to the membrane plane, respectively. The angle brackets denote time averaging. Note that the methodology relies on pressure components, which usually show huge temporal fluctuations in MD simulations.

S3 Main Transition Temperature of DPPC

Before probing the phase diagram (Fig. 1 in the main text), we first identified the main transition temperature of pure DPPC in the Slipids force field under physiological salt concentration, since this will guide us to consider the appropriate region of the phase diagram to search for the coexistence region. Four criteria were employed to determine the phase of the bilayer. These include area per lipid, deuterium order parameter profiles, lateral diffusion coefficients, as well as visual observation of the bilayers. In this section, real simulation temperatures are used, whereas in the further sections the temperatures shifted to match experimental behavior (upward shift of 6 K) based on the results in this section are employed.

S3.1 Visual Observation

The snapshots of the final structures of Chol0_{300}, Chol0_{305}, and Chol0_{310} are shown in Fig. S1**a**–**c**. These snapshots suggest that whereas the bilayer is in the ripple phase at 300 K and 305 K, the L_d phase is observed at 310 K, a few degrees below the experimental main transition temperature value of 314 K [17].

S3.2 Area Per Lipid

Here we consider the area per lipid profiles shown in Fig. S1**a**. The area of the Chol0_{310} system is 59.8 nm^2, close to values of 0.63 nm^2–0.64 nm^2 measured for pure DPPC at higher temperatures [18, 19]. The value of 0.63 nm^2 is shown in Fig. S1**a** with a dashed line. The agreement is even better when the experimental area per lipid is extrapolated linearly to 310 K following the thermal area expansivity coefficient of 0.003 K^{-1} [18]. This estimated value is shown in Fig. S1**d** with a dotted line. The slight difference with the extrapolated value and the value provided by Slipids might be due to the tendency of this force field to provide slightly too small areas per lipid for DPPC [3]. The area per lipid values calculated for the Chol0_{305} and Chol0_{300} systems show data which are close to values of 0.47 nm^2–0.48 nm^2 measured for gel phase DPPC [19, 20], in agreement with the visual observation, suggesting that they are in the ripple phase.

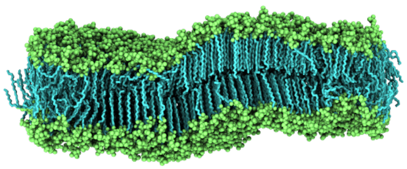

(a) 300 K

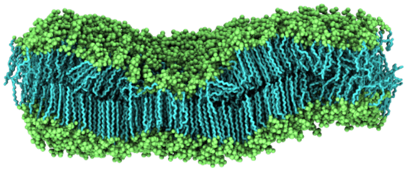

(b) 305 K

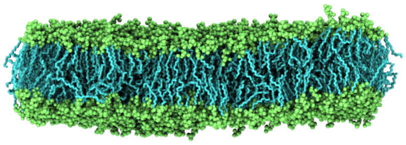

(c) 310 K

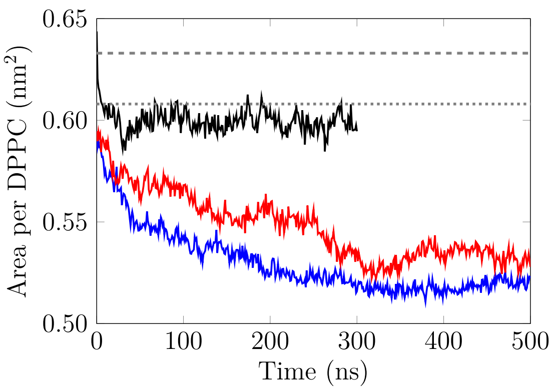

(d)

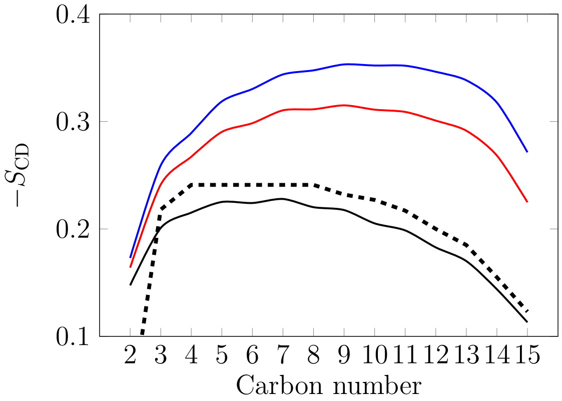

(e)

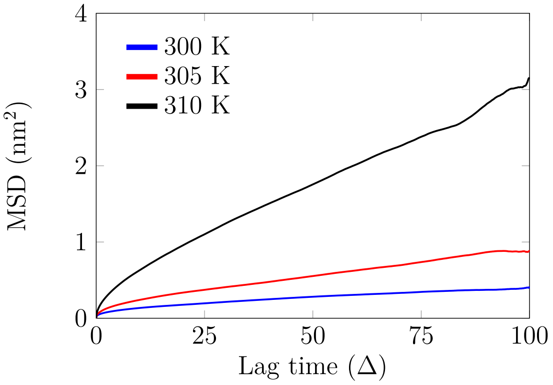

(f)

Figure S1: Results for the cholesterol-free DPPC bilayers. **a**–**c**: The final structures of the simulations of pure DPPC bilayers. **a**: Chol0_{300} **b**: Chol0_{305} **c**: Chol0_{310}. The bilayer at 310 K is in the liquid-disordered phase, the other two are in the ripple phase. Lipid chains are shown in cyan and the rest of the lipid molecules in lime. Chain hydrogens, water, and ions are omitted for clarity. **d**: Area per DPPC at various temperatures. Blue, red, and black curves correspond to systems Chol0_{300}, Chol0_{305}, and Chol0_{310}, respectively. Dashed lines show the experimental value at 323 K [18]. The dotted line is an estimation of the area per DPPC at 310 K assuming linear thermal area expansivity of 0.003 K^{-1}[18]. Color coding is given in the legend of panel **f**. **e**: Deuterium order parameter profiles. The experimentally measured profile for the L_d phase DPPC bilayer at 314 K (dashed black line) shows slightly higher values than what we obtain for our DPPC bilayer at 310 K (Chol0_{310} system). The profiles for the Chol0_{305} and Chol0_{300} systems show behavior expected for a ripple phase. Color coding is given in the legend of panel **f**. **f**: MSD curves for the pure DPPC systems. Blue, red, and black curves stand for Chol0_{300}, Chol0_{305}, and Chol0_{310} systems, respectively. Motion is measured with respect to the center of mass of the bilayer, hence the effect of drift is eliminated.

S3.3 Chain Order Parameters

The deuterium order parameter profiles along the *sn*-2 chain of DPPC molecules are plotted for pure DPPC in Fig. S1e at different temperatures. The profile of the $Chol0_{310}$ system agrees reasonably well with the experimental profile measured at 314 K [21]. The lower ordering of Slipids as compared to experiments is also apparent at 323 K as presented in the original Slipids paper [3]. The profiles for the $Chol0_{305}$ and $Chol0_{300}$ systems show significantly higher ordering, as expected for a ripple phase.

S3.4 Lateral Diffusion

The mean squared displacement (MSD) is plotted against lag time in Fig. S1**f**. The slope of this curve provides the (effective) diffusion coefficient which for the $Chol0_{310}$ is measured to be $6.4 \times 10^{-8}\text{cm}^2/\text{s}$. This value agrees with experimental values measured for L_d phase DPPC using FRAP [22], spin resonance [23], and NMR [24]. Some experiments, however, provide somewhat larger values for the diffusion coefficient. This is due to two things. First, saturated lipids in the Slipids description diffuse too slowly [3]. Second, the temperature of 310 K is smaller than that employed in experiments, and the temperature difference (310 K versus 323 K) could explain a 2.1-fold difference in the diffusion coefficients assuming an Arrhenius-type activation energy of 49 kJ/mol, a value which was measured for fluorescent probes in liquid-disordered DPPC liposomes [25]. The systems at 305 K and 300 K are in the ripple phase and have small diffusion coefficients.

S3.5 Conclusion

Based on these careful analyses, we place the main transition temperature of DPPC in the Slipids force field at ∼308 K, between 305 and 310 K, six degrees below the experimental value of 314 K for normal DPPC and 310 K for perdeuterated DPPC [17].

S4 Additional Results on DPPC–cholesterol Systems

This section contains additional results for the DPPC–cholesterol bilayers. From now on, the temperatures scaled based on the previous section will be employed, *i.e.* the simulation temperatures are incremented by 6 K to match the phase diagram presented in the main text.

S4.1 Area Per Lipid

The time dependence of the area per phospholipid is plotted in Fig. S2**a** for all cholesterol-containing systems. These profiles have converged prior to the last 100 ns employed in the analysis. Additionally, the values for the average area per phospholipid (APL) during the last 100 ns are given in Table S1.

APL is smallest for the gel phase bilayer ($Chol10_{301}$ system) for which a value of 0.50 nm^2 was obtained. Cholesterol does not condense gel phase bilayers. Therefore, we can subtract its contribution [26, 27] from the total area to estimate the area left for DPPC. This way, a value of 0.47 nm^2 is obtained, in excellent agreement with experiments on gel phase DPPC [19, 20]. The APL in the L_d phase ($Chol10_{326}$) was measured to be 0.61 nm^2, in agreement with APL of 0.63 nm^2–0.64 nm^2 measured for pure DPPC [18, 19] considering the condensing effect of cholesterol. This condensing effect is strongest at ∼20 mol% [28], resulting in APL of 0.58 nm^2 for the L_o phase ($Chol20_{316}$ system). The APL of other systems with 10 mol% cholesterol fall between these values measured for gel and L_d phase systems. The APLs of the systems with 15 mol% cholesterol are very close to those of the system with 10 mol% cholesterol, suggesting that at these cholesterol concentrations the excess area of cholesterol is negligible. All in all, the agreement with experiment is excellent.

S4.2 Radial Distribution Functions

The radial distribution functions (RDFs) of the 10th carbon along the acyl chains (both *sn*-1 and *sn*-2) are shown in Fig. S2**b**. These RDFs were calculated with the GROMACS tool `g_rdf` and the lateral distances between atoms were considered. They show that whereas in the gel phase (*e.g.* the $Chol10_{301}$ system) the

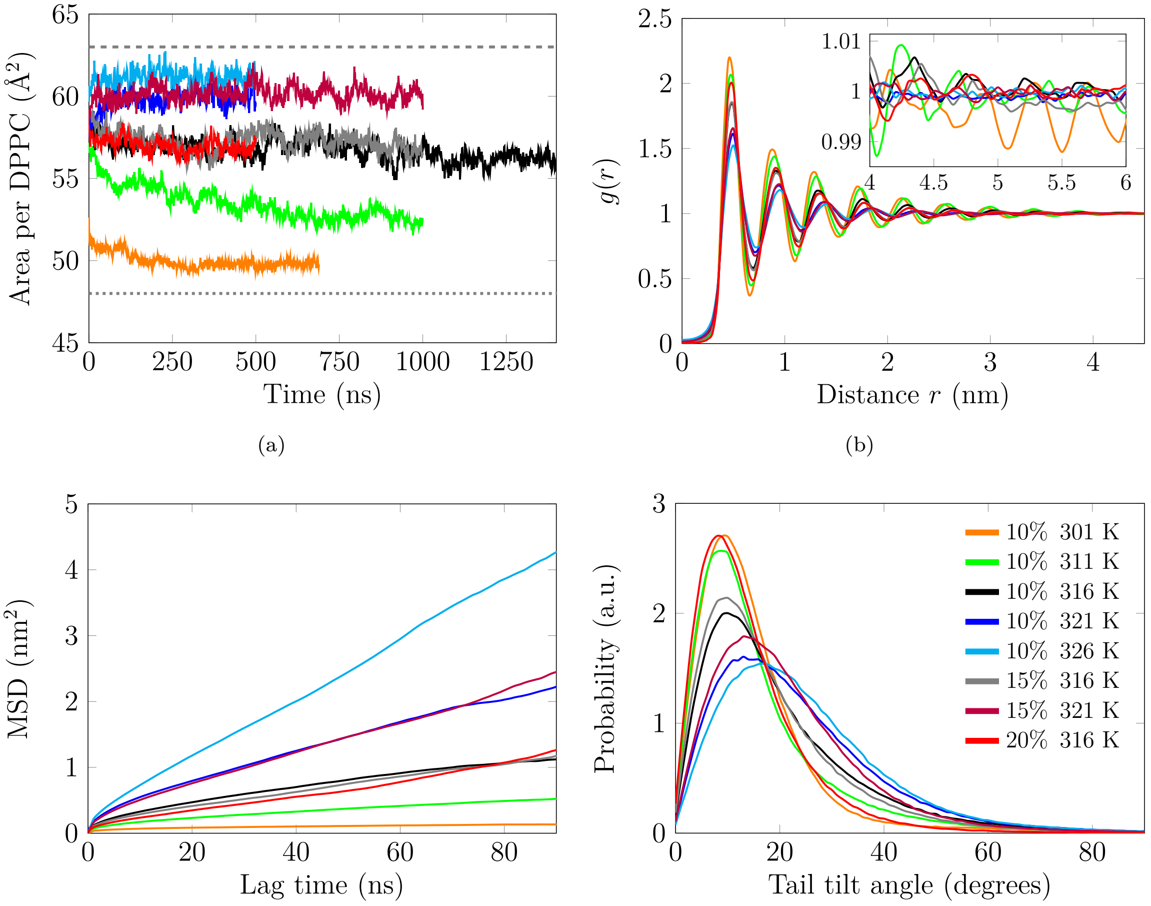

(a) (b) (c) (d)

Figure S2: Results for cholesterol-containing systems. All plots follow the same color coding shown in panel **d**. **a**) Areas per phospholipid in the simulated systems containing cholesterol. Dashed and dotted gray lines show experimentally measured area per lipid in the fluid and gel phases of pure DPPC (∼0.63 and ∼0.48 nm^2, respectively). **b**) Lateral radial distribution functions of the 10th carbons along the DPPC acyl chains. Inset shows a zoomed-in version of the data between 4 and 6 nm. **c**) Lateral mean squared displacements of DPPC molecules in the membrane plane. **d**) Distributions of the lipid chain tilt angles.

chain positions are correlated over the whole simulation box (see inset in Fig. S2**b**), this correlation dies off in the liquid-disordered phase (*e.g.* the Chol10$_{326}$ system) in approximately 3 nanometers. The liquid-ordered Chol20$_{316}$ system as well as the Chol10$_{316}$ system with its heterogeneity fall between these behaviors, as expected.

S4.3 Mean Squared Displacement

Despite the computational limitations explained in Section S2.1, the curves presented in Fig. S2**c** show that diffusion is two orders of magnitude faster in the Chol10$_{326}$ system (L_d phase) compared to the gel phase (Chol10$_{301}$) system. The effective diffusion coefficients are obtained from a linear fit to the MSD data versus lag time (Δ) as $D = \lim_{\Delta\to\infty} \mathrm{MSD}/4\Delta$. The lag time interval between 10 and 90 ns was used for fitting. We obtain values of 0.2×10^{-8} cm^2/s and 11.2×10^{-8} cm^2/s for these gel (Chol10$_{301}$) and L_d phase (Chol10$_{321}$) systems, respectively. The value of the gel phase is higher than expected and it results likely from the thin regions (see Section S4.5). The Chol20$_{316}$ system (L_o phase) shows intermediate behavior between the gel and liquid-disordered systems with an effective diffusion coefficient of 3.1×10^{-8} cm^2/s. The system displaying heterogeneity (Chol10$_{316}$) has a quite small value of 2.5×10^{-8} cm^2/s, in line with the low mobility of the hexagonally packed core of the nanodomain.

S4.4 Lipid Tilt Angle

The gel phase of a lipid membrane can take various forms and some of them are associated with tilted all-trans lipid chains with characteristic tilt angles. On the other hand, the chains in a liquid-ordered bilayer do not show such collective tilting. Therefore, the lipid chain tilt angle can provide information on the presence of a particular gel phase. The tilt angles of the lipid chains were calculated as the angle between the z axis and the vector between the carbonyl carbon and the terminal carbon of the acyl chain. Both *sn*-1 and *sn*-2 chains of the lipids in one leaflet were considered in the analysis.

These tilt angle distributions are shown in Fig. S2**d**. The chain tilting in the gel-phase systems (Chol10$_{301}$ and Chol10$_{311}$) is very similar to that in the L_o phase system (Chol20$_{316}$) with their maxima located at ∼10 degrees. The two L_d phase systems at higher temperatures (Chol10$_{321}$ and Chol10$_{326}$), on the other hand, show qualitatively very different behavior and a higher average tilt of ∼20 degrees. The heterogeneous system (Chol10$_{316}$) shows characteristics of these two classes. The maximum of the distribution is reached at an angle close to 10 degrees, indicating high order, yet the chain also extends to higher chains tilts characteristic of the disordered phase. It is noteworthy that the gel phase is a straight-chained one and a single tilt angle characteristic for the tilted gel phase is not observed. This characteristic angle is also invisible in the distribution for the Chol10$_{316}$ system.

S4.5 Thickness Maps

All thickness maps are shown in Fig. S3. The thickness maps for the gel phase systems (Chol10$_{301}$ and Chol10$_{311}$) reveal the existence of thinner regions. These regions correspond to those of higher mobility (see Fig. S5) and result from the inability of the system with limited size and periodic boundary conditions to relax into a uniform gel phase. A closer look at the gel phase structure reveals that ripples, chain interdigitation, and chain tilting are all present in these two gel-phase systems (data not shown).

The local thickness of the Chol10$_{326}$ system is approximately 4 nm or even below. This behavior agrees with X-ray [19] and NMR [29] measurements on L_d phase DPPC bilayers, which provide thickness values of 3.83 and 3.89 nm, respectively. The thickness map of the Chol20$_{316}$ system shows a fairly uniform distribution of thicknesses around 4.5 nm. This value is in agreement with the experiments on DPPC–cholesterol bilayers in the L_o phase. With 25 mol% cholesterol, a value of 4.61 nm was obtained at 325 K using neutron scattering techniques [30]. A similar value of 4.49 nm was measured also with NMR at 318 K [29]. A domain with higher thickness is clearly present in the Chol10$_{316}$ system, whereas the thickness outside this domain corresponds to that of an L_d phase. The inverse is observed in the Chol15$_{316}$ system as small disordered domains are observed in the mostly ordered system. This likely results from a limited system size. The Chol15$_{321}$ system shows some heterogeneity, yet the contributions of the ordered and disordered regions probably flatten due to the dynamic nature of the lipids in this system.

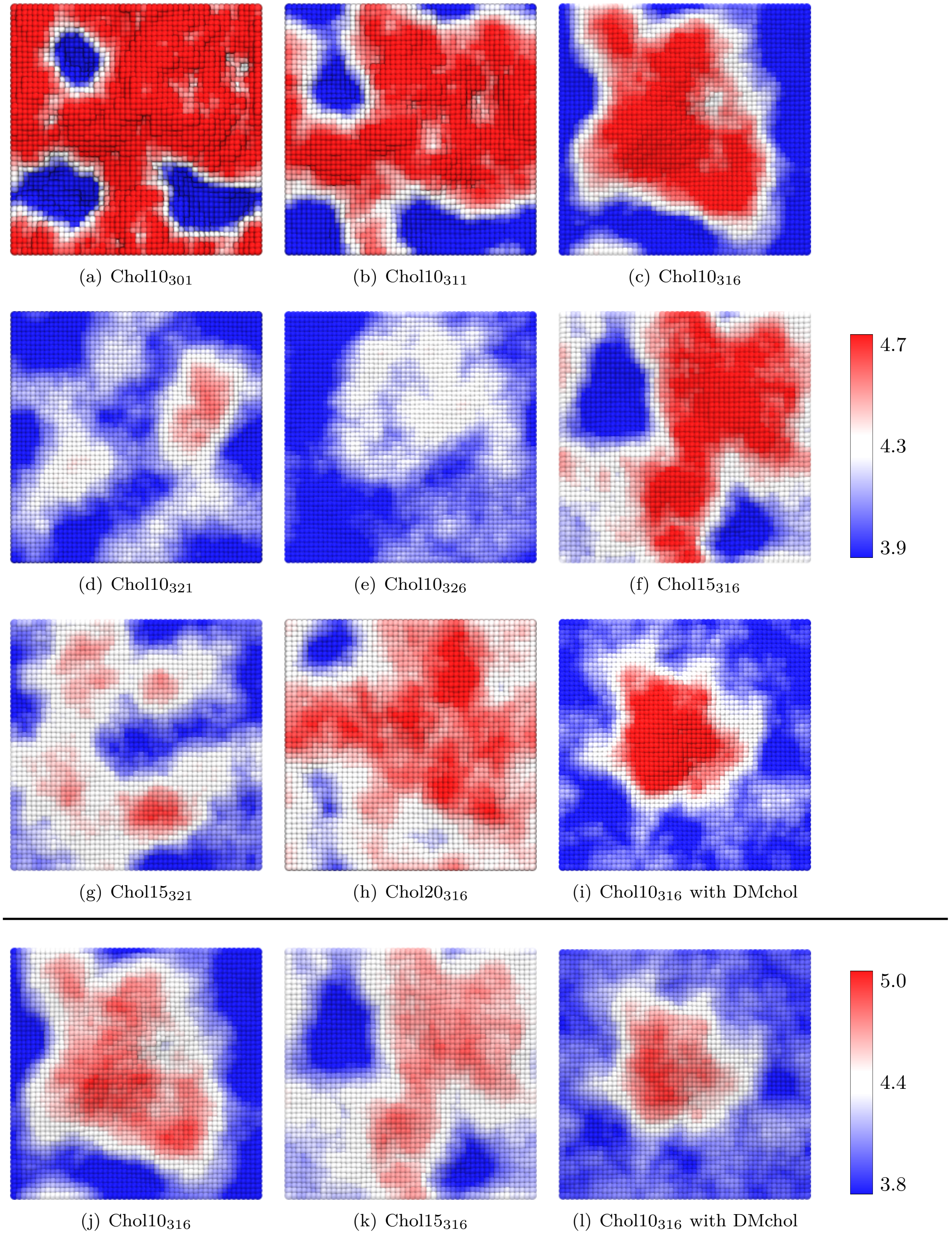

(a) Chol10$_{301}$ (b) Chol10$_{311}$ (c) Chol10$_{316}$

(d) Chol10$_{321}$ (e) Chol10$_{326}$ (f) Chol15$_{316}$

(g) Chol15$_{321}$ (h) Chol20$_{316}$ (i) Chol10$_{316}$ with DMchol

(j) Chol10$_{316}$ (k) Chol15$_{316}$ (l) Chol10$_{316}$ with DMchol

Figure S3: Thickness maps. For the bottom row, the color scale is adjusted to show the domain substructure in more detail.

The bottom row in Fig. S3 shows a more detailed thickness map of selected systems. Data are shown for $Chol10_{316}$ and $Chol15_{316}$ systems to reveal the substructure of the ordered domain. They indeed indicate that the domain in the $Chol10_{316}$ system has very thick (dark red) areas corresponding to the hexagonal packing of lipid chains. Such very thick regions are absent in the thickness map of the $Chol15_{316}$ system with most of the system showing values characteristic of the L_o phase (light red and white), in agreement with the order parameter distributions shown in the main text. The highest thickness values present in the $Chol10_{316}$ system are approximately 5 nm. This is below the value of 5.5 nm measured using AFM for gel phase DPPC bilayer at 295 K [31]. Additionally, collective tilting of all lipid chains in the gel-like region, which could explain the smaller thickness values, was not observed. Therefore, we prefer not to refer to these hexagonally packed regions as being gel but rather consider that they are a part of the L_o phase, in agreement with recent studies [32].

Additionally, data for the system with DMchol are shown on the right bottom panel of Fig. S3. In this system ordered regions (light red and white) are not present to a large extent and the system consists of disordered regions (blue) and a hexagonally packed (dark red) domain. As discussed in the main text, this hexagonally packed domain is likely to dissolve during further simulation.

S4.6 Deuterium Order Parameter Distributions

The deuterium order parameter distributions along the *sn*-2 chains of DPPC are shown in Fig. S4 for all systems, including those omitted in the main paper. The $Chol10_{311}$ system is mostly in gel phase, yet it exhibits some disordered as well as ordered behavior as well. The disordered nature is most likely due to the structural limitations posed by rippling and other non-uniform structures present in the studied gel phases systems as discussed below. The DPPC molecules interacting with cholesterols are expected to show liquid-ordered behavior according to the phase diagram. The $Chol10_{321}$ is generally in the L_d phase, yet some lipids also show more ordered values. The distribution for the $Chol15_{316}$ systems shows both ordered and disordered lipid chains, while that of the $Chol15_{321}$ system shows intermediate behavior which is linked to lipid chains switching between ordered and disordered states as described in the main text.

S4.7 Average Displacement Maps

The average in-plane lipid displacement maps are shown in Fig. S5. The $Chol10_{326}$ system shows overall high mobility characteristic for the L_d phase. The $Chol10_{301}$ system on the other hand is practically frozen, indicating gel-like behavior. The mobility of lipids in the $Chol20_{316}$ system falls between these two, which is expected for a system in the L_o phase. The $Chol10_{316}$ and $Chol15_{316}$ systems again combine the behaviors of the single phase systems as they contains regions of higher and lower mobilities. In addition to L_o-like and L_d-like behavior, some really immobile lipids are found within a domain of lesser mobility in the $Chol10_{316}$ system. These gel-like features agree with the earlier result that instead of just heterogeneity with L_o-like and L_d-like behavior, a third component might be present in this system. This immobile region is absent in $Chol15_{316}$, indicating the absence of the gel component in this system as suggested by the fits to the deuterium order parameter distributions explained in the main text. This is also evident from Fig. S5j, which shows a histogram of the spatially resolved displacement. Here, unlike in the $Chol10_{316}$ system, the practically immobile lipids are not observed in the $Chol15_{316}$ system. The $Chol15_{321}$ system also shows slight heterogeneities in lipid motion, yet the separation into different regions is not very evident. Other displacement maps also show behavior that is in line with these observations.

S4.8 Order Parameter Correlation between Leaflets

The spatial correlation of the average deuterium order parameter of the *sn*-2 chain between the two membrane leaflets is shown in Fig. S6.

Whereas the order parameter values show narrow distributions for other systems representing L_d, L_o and gel phases, the $Chol10_{316}$ and $Chol15_{316}$ systems show a much wider range of order parameter values. In the $Chol10_{316}$ system two maxima exist. The location of one of these two maxima corresponds clearly to that of the L_d system, *i.e.* $-S_{CD} \sim -0.2$. The other one is located at higher values characteristic for both L_o and gel phases. In the $Chol15_{316}$ system the maximum corresponding to L_o is present to much higher

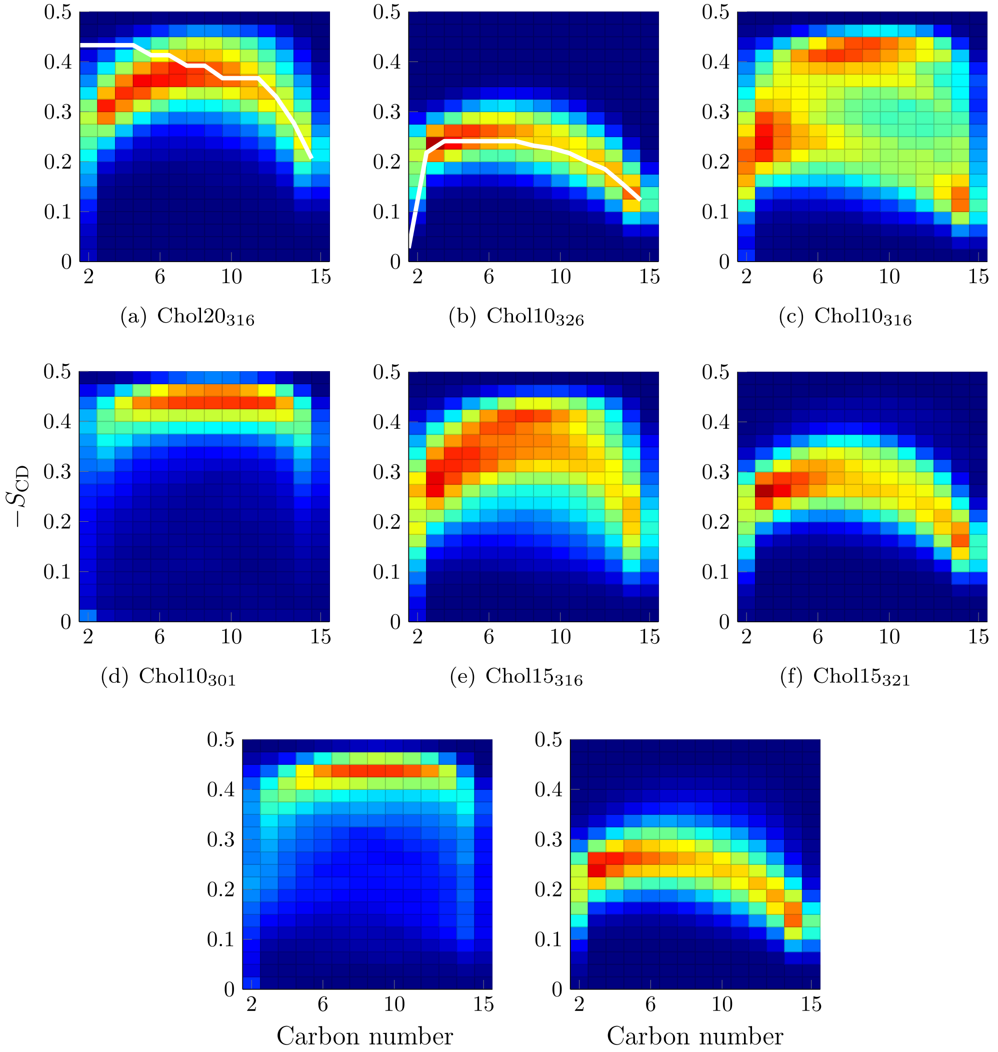

(a) Chol20_{316} (b) Chol10_{326} (c) Chol10_{316}

(d) Chol10_{301} (e) Chol15_{316} (f) Chol15_{321}

(g) Chol10_{311} (h) Chol10_{321}

Figure S4: Order parameter distributions for all systems. Available experimental data are shown in white (full line). For the Chol20_{316} system, given here are the data measured for the L_o phase (DPPC + 40 mol-% cholesterol at 308 K) [33]. For the Chol10_{326} system (5), the experimental data are for the L_d phase system (pure DPPC at 314 K) [21]. The deviation at the beginning of the curve is discussed in the main paper.

extent in agreement with the estimations of the phase composition of the systems given in the main text. The lipid chains in the Chol15_{321} system show the maxima at an intermediate position indicating that they dynamically switch between ordered and disordered conformations.

This analysis also provides information on the spatial correlation of the order parameters between membrane leaflets. Based on the diagonal positioning of the values in the plot for the Chol10_{316} and Chol15_{316} systems, the ordered regions in one leaflet coincide with the ordered regions in the other leaflet and this also holds for the disordered regions. This indicates that even this simple two-component model system reproduces the bilayer registry effect, *i.e.*, the inter-leaflet correlation of lipid order.

S4.9 Line Tension Calculation

It is crucial for the validity of the line tension values that the domain boundary is stable along the predetermined axis. Visual observation of the final structures of the line tension calculations (see Section S2.6), shown

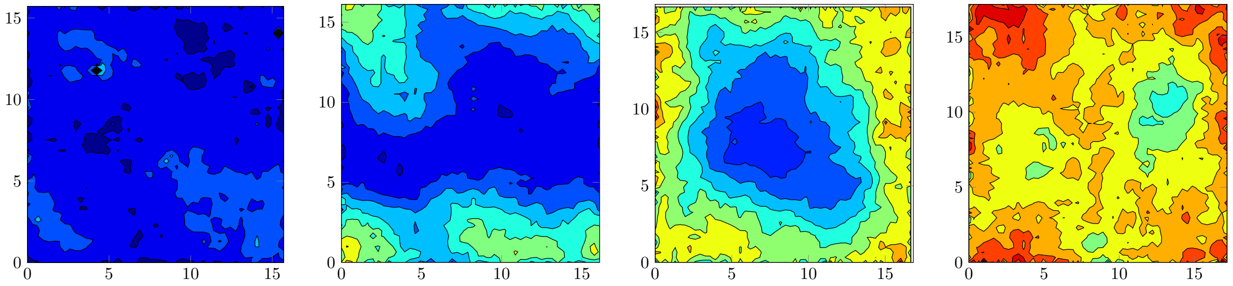

(a) Chol10_{301} (b) Chol10_{311} (c) Chol10_{316} (d) Chol10_{321}

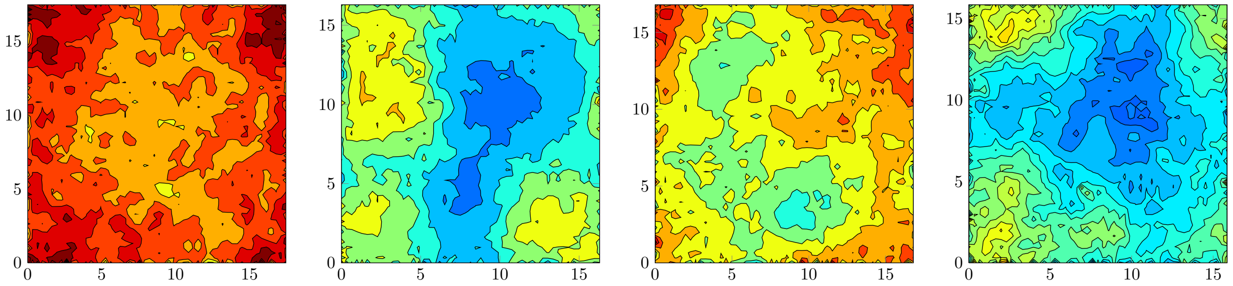

(e) Chol10_{326} (f) Chol15_{316} (g) Chol15_{321} (h) Chol20_{316}

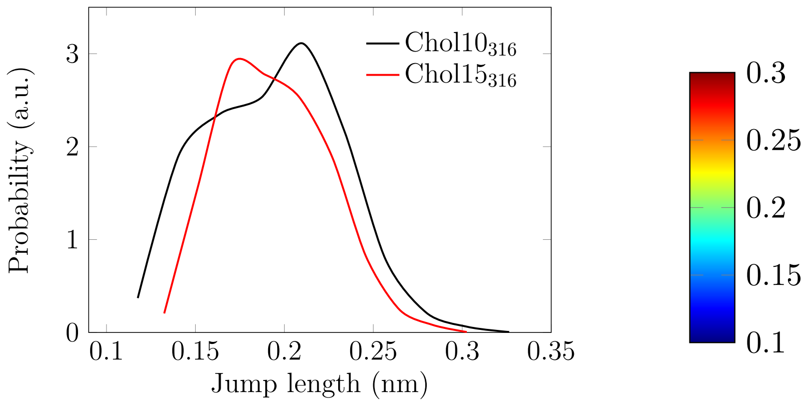

(i)

Figure S5: **a**–**h**: Average displacement of DPPC center of mass during a 1 ns interval. **i**: Histograms of the average in-plane lipid displacement extracted from **c** and **f**. The probabilities of having very small displacements (∼0.1–0.15 nm) are very different in Chol10_{316} and Chol15_{316} system, indicating that the almost immobile hexagonal core of the nanodomain is not present in the latter system.

in Figs. S7**a** and **b** suggests that this is indeed the case for the binary DPPC–cholesterol and the ternary DOPC–DPPC–cholesterol systems. This is however not true for DMchol (see Section S4.13). We also ensured the stability of the domain boundaries in the line tension calculations quantitatively in two ways. First, the mass densities of the lipid molecules including cholesterol across the domain boundary were calculated. Similarly to the actual line tension calculation, the first 300 ns of simulation were discarded and the data for the subsequent 300 ns were analyzed. These density profiles are shown in Figs. S7**c** and **d** for the binary and ternary systems, respectively. These plots show that the components have not mixed during the simulations, yet the boundaries are fairly diffusive in both systems.

Additionally, for the binary system the deuterium order parameter profiles for the DPPC *sn*-2 chain were calculated across the domain boundary. These profiles, plotted in Fig. S7**e**, show that the ordered–disordered

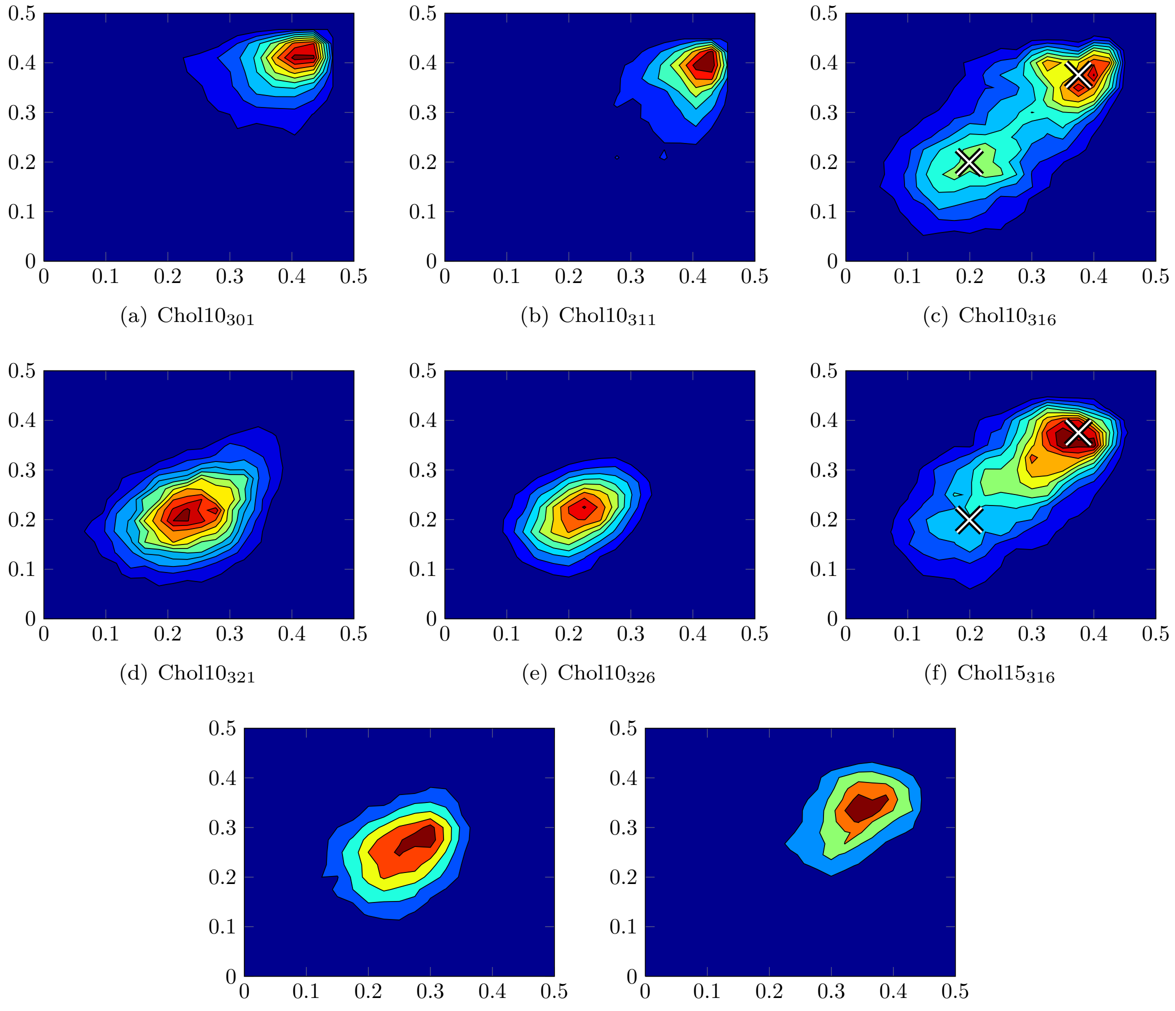

(a) $\mathrm{Chol10}_{301}$ (b) $\mathrm{Chol10}_{311}$ (c) $\mathrm{Chol10}_{316}$

(d) $\mathrm{Chol10}_{321}$ (e) $\mathrm{Chol10}_{326}$ (f) $\mathrm{Chol15}_{316}$

(g) $\mathrm{Chol15}_{321}$ (h) $\mathrm{Chol20}_{316}$

Figure S6: The spatial correlation of average deuterium order parameter of the *sn*-2 chain between the membrane leaflets. The axes show data for the two leaflets. The crosses drawn in the plot for the $\mathrm{Chol10}_{316}$ and $\mathrm{Chol15}_{316}$ systems show average values calculated from the experimental data for $\mathrm{L_o}$ (DPPC + 40 mol % cholesterol at 308 K [33]) and $\mathrm{L_d}$ (DPPC at 315 K, [21]) phases.

boundary is stable throughout the simulation as was expected from the density profiles.

The cumulative averages of line tension for binary and ternary systems (see Section S2.6) are shown in Fig. S7**f**. It is evident that even though the curves show large fluctuations, the line tension associated with the domain boundary in the ternary mixture (∼60 pN) is an order of magnitude larger than that in the binary one (∼5 pN).

Due to the scarcity of experimental data, direct comparison to experiments is challenging. Measurements on ternary mixtures of sphingomyelin–DOPC–cholesterol provided line tension values around 1 pN [34] whereas the line tension of the gel–liquid domain boundaries in the DOPC–DSPC system was measured to be approximately 4 pN [35]. These values are somewhat different to our estimates. However, a value of 14.4 pN was measured from a simulation study on ternary systems employing the coarse-grained Martini force field [36], indicating that the quantitative agreement between experiments and simulations might be poor in general. Despite this, the order of magnitude difference in our values between the binary and ternary systems is qualitatively significant and consistent with experiments.

It must however be noted that the mixing entropies and other free energy terms in the binary and ternary

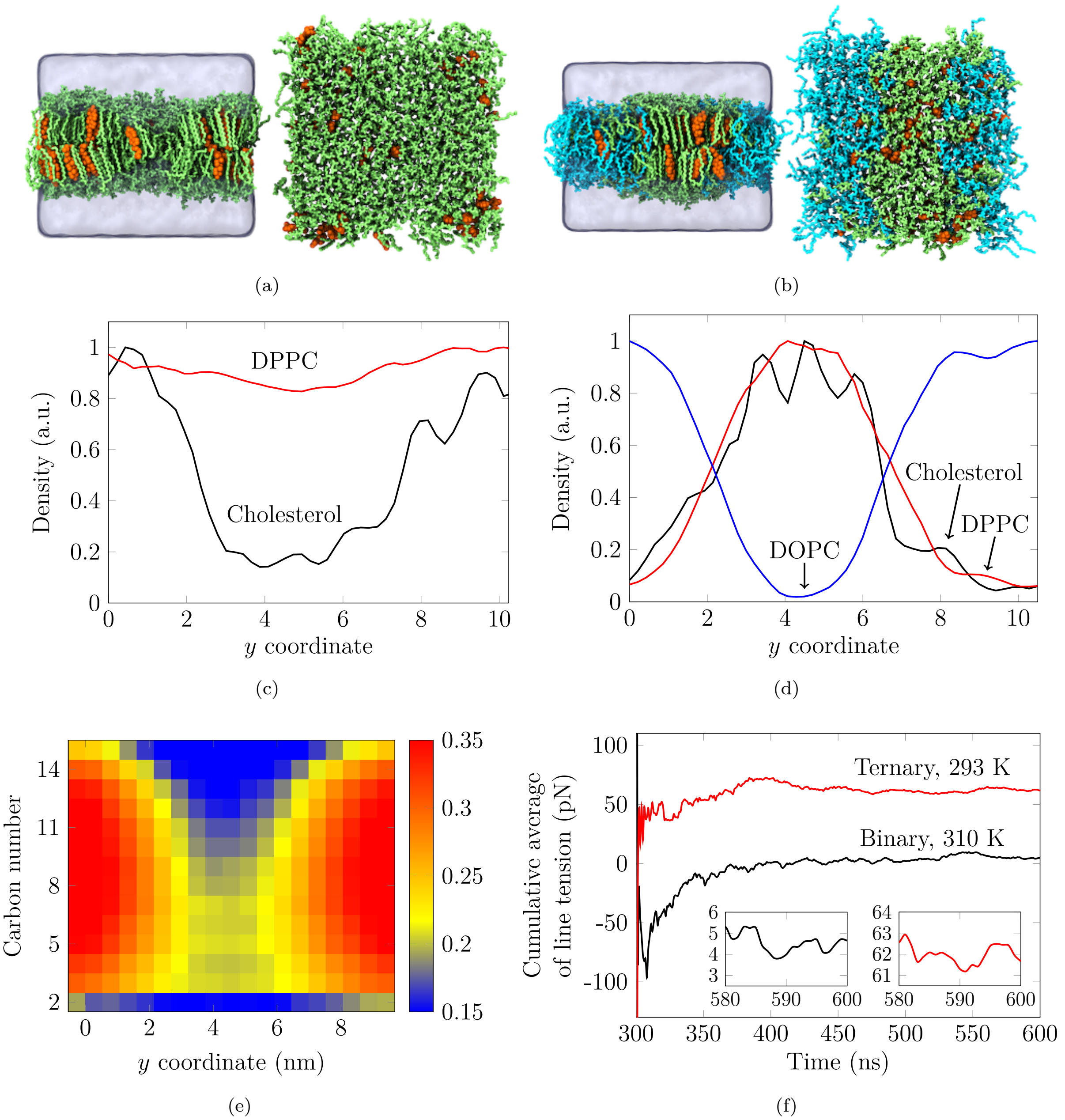

Figure S7: Results of the line tension calculation. **a** & **b**: Final structures (after 600 ns) of the binary (**a**) and ternary (**b**) lipid mixtures employed in the line tension calculation. DPPC is colored in green, DOPC in cyan, and cholesterol in orange. Water is depicted as transparent blue. **c** & **d**: Density profiles of lipid components in the systems employed for the line tension calculation. **c**: Binary DPPC–cholesterol system at 310 K (simulation temperature, 316 K effective temperature). **d**: Ternary DOPC–DPPC–cholesterol system at 293 K (simulation temperature). Profiles are normalized so that maximum density is 1 for all profiles. **e**: Deuterium order parameter along the *sn*-2 chain of DPPC in the binary system. The domain boundaries are positioned perpendicularly to the y coordinate as evidenced by the strong variation in the order parameter values along the y coordinate. **f**: Cumulative averages of line tension. The insets show the last 20 ns of the data in more detail. Note that real simulation temperatures are given here.

systems are different. This, together with the lack of consensus on the relevant energy terms [14], prevent us from estimating the domain sizes based on these calculated values only.

S4.10 Thickness Distributions

The thickness distributions are shown in Fig. S8**a** for the $\mathrm{Chol10}_{316}$ system in Fig. S8**b** for the $\mathrm{Chol15}_{316}$ system. Each plot can be fitted by two Gaussian functions whose mean values (4.0 nm and 4.6 nm) are characteristic for liquid-ordered and liquid-disordered phases, as expected. For other systems, a single Gaussian fits the data well. We have also calculated the thickness distributions only for the regions where cholesterol is present. The curves calculated for cholesterol in the $\mathrm{Chol15}_{316}$ system (dashed lines in Fig. S8**b**) are very similar to those obtained for the $\mathrm{Chol10}_{316}$ system in Fig. S8**a**, indicating that cholesterol resides in the ordered regions. The data for the whole membrane (solid lines in Fig. S8**b**), however, show a very different distribution compared to that for the $\mathrm{Chol10}_{316}$ system in Fig. S8**a**. The distribution is clearly shifted towards larger thickness values as expected for a system with a larger contribution from the ordered regions. The highest thickness values are absent indicating that the $\mathrm{Chol15}_{316}$ system does not contain the hexagonally packed regions.

Similar conclusions can be drawn from Fig. S8**c**, which shows the distribution of the norm of the thickness gradient in the membrane plane. By comparing the shapes of the distributions obtained for the whole membrane and for those obtained for locations occupied by cholesterol, we can hypothesize two things: First, cholesterol does not tend to locate itself to the exact domain boundaries where the thickness is assumed to change very rapidly, as the probability at higher values of the histogram is small. Second, cholesterol is also excluded from the planar regions located at values close to zero in the gradient histogram.

S4.11 Nearest Neighbor Distributions

Information on the packing of the ordered domain can be obtained from the nearest neighbor distributions calculated as explained in Ref. 10. These nearest neighbor distributions are plotted in Fig. S9.

Whereas lipid chains in the gel phase ($\mathrm{Chol10}_{301}$ system) often have six neighbors (sometimes even seven or eight) in the first coordination shell as expected for the hexagonal packing, the probability of having six neighbors drops down significantly in the disordered $\mathrm{Chol10}_{326}$ system. In the heterogeneous systems ($\mathrm{Chol10}_{316}$ and $\mathrm{Chol15}_{316}$), the distributions show that some hexagonally packed regions are present in the system. Further conclusions can be drawn from a comparison of the neighbor distributions calculated with and without cholesterol molecules included in the analysis. These distributions, plotted in the rightmost bottom panel of Fig. S9 for the $\mathrm{Chol10}_{316}$ system, show that cholesterol has little effect on the tight-packed clusters of lipid chains, yet it perturbs the distributions in the second coordination shell substantially.

S4.12 Location of Cholesterol Flip-flops

The transbilayer movement of cholesterol might act as a stabilizer for height mismatch associated with membrane domains [37]. Therefore the flip-flops must take place rapidly in phase-separated systems. Cholesterol partitions mostly to the liquid-ordered phase. The rate of cholesterol flip-flops in this phase is, however, orders of magnitude slower than that in the $\mathrm{L_d}$ phase [38]. To solve this discrepancy, we monitored the locations of the spontaneous flip-flops in our system with heterogeneity (the $\mathrm{Chol10}_{316}$ system) and observed a total of seven events during the 1.4 μs simulation. The flip-flops were associated to the position and moment at which the cholesterol had reoriented itself to the new leaflet. The membrane thickness maps at the time of flip-flop were calculated by considering data from 15 ns before until 5 ns after the moment of a given flip-flop. These thickness maps are shown in Fig. S10 together with the cholesterol flip-flop locations. It is clear that the cholesterol molecules flip at the domain boundaries. More specifically, these flip-flops take place on the disordered side of these boundaries. This is probably due to the structural defects at this region caused by the domain height mismatch. The limited number of observed spontaneous flip-flop events, however, calls for a more systematic study of the issue. In addition, in real cellular membranes the role of proteins cannot be neglected [39].

Cholesterol flip-flop has been suggested to decrease the free energy associated with the mismatch of transbilayer domain coupling, yet this effect is estimated to be small [37] with dynamic chain interdigitation

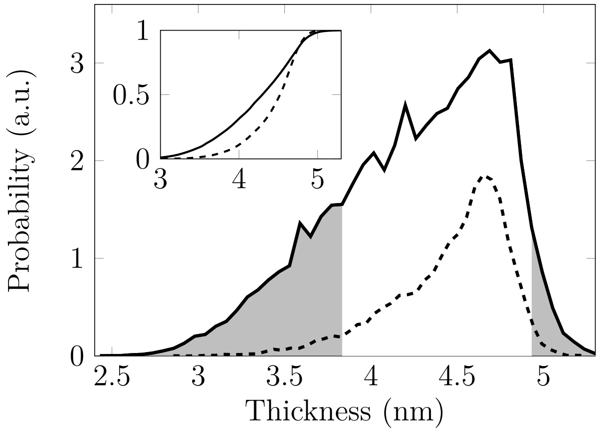

(a)

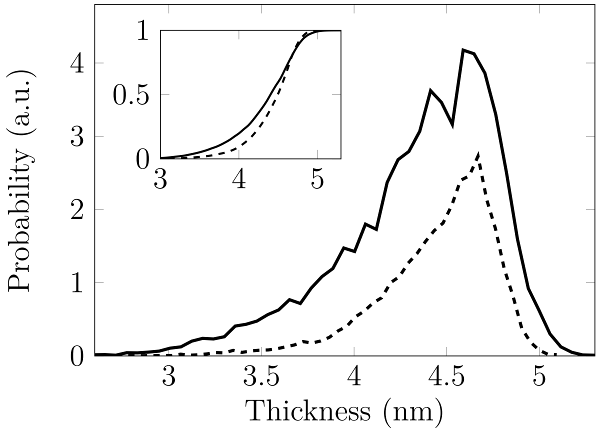

(b)

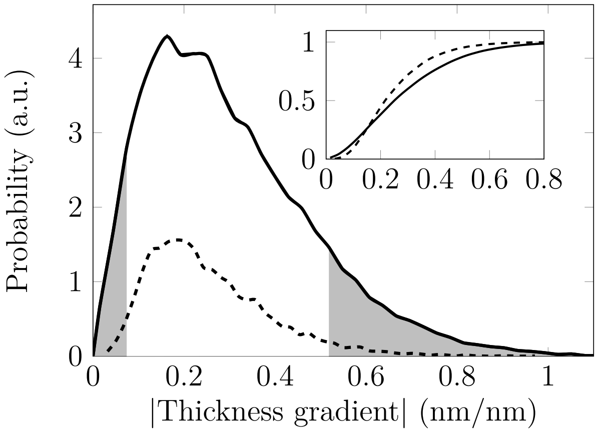

(c)

Figure S8: Thickness and thickness gradient histograms. **a**: Thickness histogram for the Chol10_{316} system. **b**: Thickness histogram for the Chol15_{316} system. In (**a**) and (**b**) the inset shows the normalized cumulative distributions. Solid lines show data collected from the whole system and dashed lines data collected from locations occupied by cholesterol. Gray fill highlights regions with a small local concentration of cholesterol. **c**: Histogram of the norms of the gradient of membrane thickness. Solid lines show data collected from the whole system and dashed lines data collected from locations occupied by cholesterol. The inset shows normalized cumulative histogram of the data and gray fill highlights regions with small presence of cholesterol.

playing a larger role in the stabilization process [40]. Therefore it is quite intriguing that the flip-flops seem to occur at the boundaries of ordered domains as rapid transbilayer motion of cholesterol in these regions could efficiently negate the energetic penalty associated with the mismatch. One reason why lipid rafts are nanoscopic cholesterol-containing domains might therefore be to maximize the boundaries associated with them and therefore maximize the transbilayer mobility of cholesterol. The flip-flop rates could be further enhanced in these regions by the presence of cholesterol-binding transmembrane proteins with phospholipid flipping abilities that further perturb the local lipid composition and promote transbilayer mobility [41, 42].

S4.13 Effects of Cholesterol Ring Demethylation

Results characterizing the DMchol system are shown in Fig. S11. The area per lipid profile (Fig S11**a**) seems to stabilize after ∼550 ns, justifying the use of the last 100 ns (600–700 ns) for analyses. Further, the condensing effect of DMchol is weaker than that of cholesterol, in line with both experiments and simulation

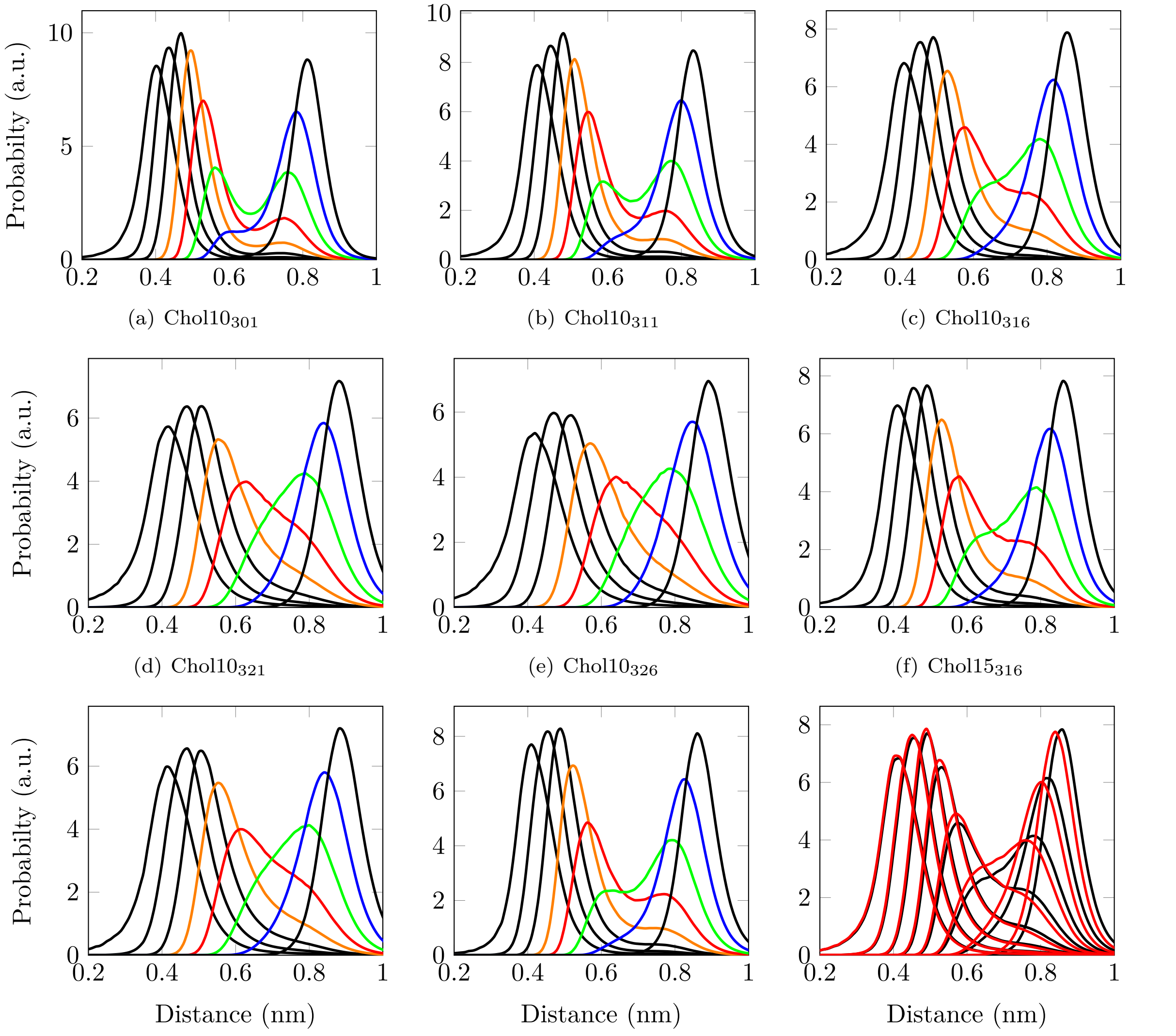

(a) Chol10$_{301}$ (b) Chol10$_{311}$ (c) Chol10$_{316}$

(d) Chol10$_{321}$ (e) Chol10$_{326}$ (f) Chol15$_{316}$

(g) Chol15$_{321}$ (h) Chol20$_{316}$ (i) Chol10$_{316}$ *

Figure S9: Nearest neighbor distributions. The curves for 4th to 7th carbons are colored in orange, red, green, and blue whereas the curves for first three and the eighth carbons are shown in black. In the bottom right panel (*), results performed with (red) and without (black) cholesterol included in the analysis are shown.

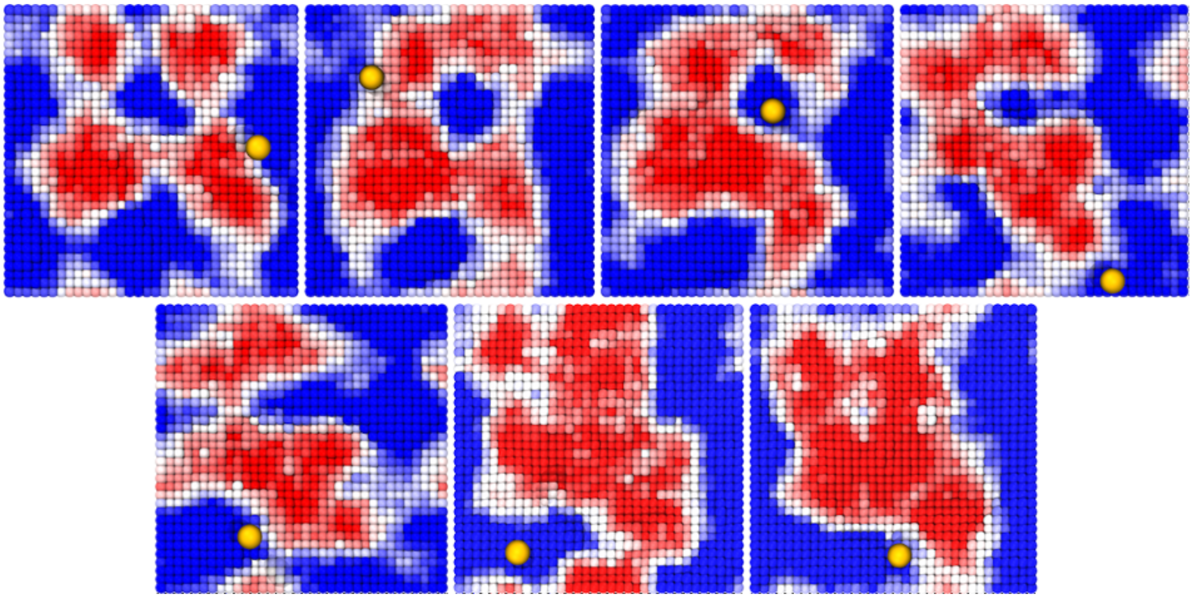

Figure S10: Locations of the seven observed spontaneous cholesterol flip-flops (orange spots) with respect to the local thickness of the system obtained similarly as for the thickness maps (Fig. S3). The events occur at 210 ns, 335 ns, 390 ns, 760 ns, 795 ns, 1180 ns, and 1250 ns of simulation time.

studies [2, 43, 44]. The deuterium order parameter distribution (Fig S11**b**) shows that in comparison to cholesterol, DMchol has a weaker ordering effect on the system. A small gel-like contribution is present, yet the behavior characteristic for the L_o phase seems to be mostly absent. Therefore the thickness map for the DMchol system (shown in Figs. S3**i** and **l**) most likely contains two components: thin disordered regions surrounding a thick hexagonally packed domain in the center. As mentioned in the main text, this hexagonally packed domain is expected to dissolve eventually.

We also attempted to calculate the line tension of the binary system after replacing cholesterol with DMchol. However, we observed that the domain boundary was not stable, in line with the observed dissolution of the spontaneously formed nanodomain after cholesterol was replaced with DMchol (see Fig. S3**i**). The unstable domain boundary is evidenced by Fig. S11**c**, which shows the density profiles of DPPC and DMchol in direction perpendicular to the domain boundary. The DMchol density shows a fairly flat profile, which is in agreement with visual observation suggesting that DMchol is able to diffuse out from the original ordered half of the simulation box, invalidating the analysis. Altogether, these findings suggest that in a binary mixture of DPPC and DMchol, there is no heterogeneous region of the same type as with DPPC and cholesterol above the main transition temperature.

S5 Comparison with Studies Employing Other MD Models

The capability of the Slipids force field to display ordered liquid nanodomains is quite peculiar. Since the coarse-grained Martini force field does not predict this behavior [45–47], it would be the first assumption to relate this discrepancy to the differences in the saturated chains of DPPC. As these chains are presented in Martini by only four beads each, at atomistic resolution they have many more degrees of freedom. This allows them to display a much wider range of configurations both when interacting with other DPPC molecules or when in contact with cholesterol. Another possibility is that the cholesterol molecules in the Martini model do not have a rough and a smooth face which might be important for domain formation [32, 48]. These two possibilities are, however, challenged by the results of Waheed et al. [45]. Their united-atom Berger force field contains near-atomistic level description of the lipid chains (with methyl and methanediyl groups described with one particle) and cholesterol with two distinguishable faces. Still, this model does not suggest the

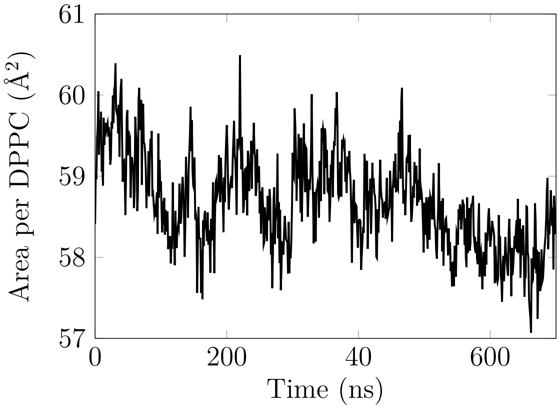

(a)

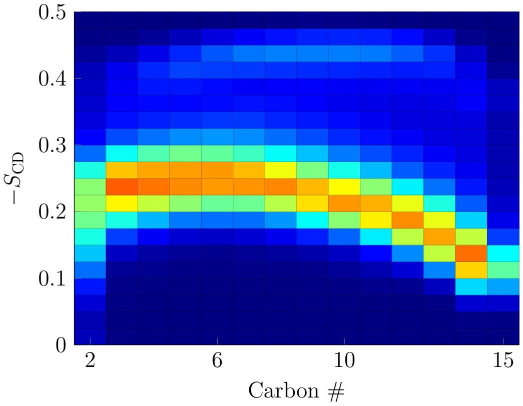

(b)

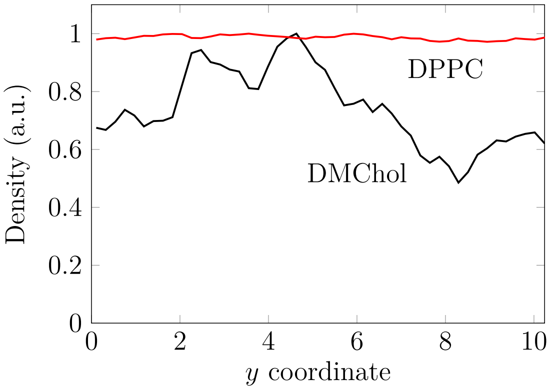

(c)

Figure S11: Additional results for the DMchol systems. **a**) Time evolution of area per phospholipid. **b**) Order parameter distribution along the *sn*-2 chain of DPPC during the last 100 ns of the simulation. **c**) Density profiles of lipid components in the system with demethylated cholesterol employed in the line tension calculation. Profiles are normalized so that maximum density is one for all profiles.

formation of nanoscale ordered domains. This might be due to the size and simulation time constraints which prevent the formation of domains. The aforementioned united-atom systems contained 1024 lipids which is very close to our number of 1152. However, the authors do not mention the duration of their simulation. Based on our results, sub-microsecond time scales are not sufficient to adequately probe domain formation. Also, the inability of the original Berger model to properly describe the phase behavior of DPPC might result in disagreement with our findings [49]. The Slipids force field, on the other hand, properly captures the phase behavior of pure DPPC except for the slight difference in the main transition temperature [3].

References

[1] Vist, M. R.; Davis, J. H. *Biochemistry* **1990**, *29*, 451–464.

[2] Róg, T.; Pasenkiewicz-Gierula, M.; Vattulainen, I.; Karttunen, M. *Biophys. J.* **2007**, *92*, 3346–3357.

[3] Jämbeck, J. P.; Lyubartsev, A. P. *J. Phys. Chem. B* **2012**, *116*, 3164–3179.

[4] Jämbeck, J. P.; Lyubartsev, A. P. *J. Chem. Theory Comput.* **2012**, *9*, 774–784.

[5] Jorgensen, W. L.; Chandrasekhar, J.; Madura, J. D.; Impey, R. W.; Klein, M. L. *J. Chem. Phys.* **1983**, *79*, 926–935.

[6] Darden, T.; York, D.; Pedersen, L. *J. Chem. Phys.* **1993**, *98*, 10089.

[7] Bussi, G.; Donadio, D.; Parrinello, M. *J. Chem. Phys.* **2007**, *126*, 014101.

[8] Parrinello, M.; Rahman, A. *J. Appl. Phys.* **1981**, *52*, 7182–7190.

[9] Hess, B.; Kutzner, C.; van der Spoel, D.; Lindahl, E. *J. Chem. Theory Comput.* **2008**, *4*, 435–447.

[10] Mazur, S. *J. Chem. Phys.* **1992**, *97*, 9276–9282.

[11] Jeon, J.-H.; Monne, H. M.-S.; Javanainen, M.; Metzler, R. *Phys. Rev. Lett.* **2012**, *109*, 188103.

[12] Gapsys, V.; de Groot, B. L.; Briones, R. *J. Comput. Aid. Mol. Des.* **2013**, *27*, 845–858.

[13] MATLAB, *version 8.2.0 (R2013b)*; The MathWorks Inc.: Natick, Massachusetts, 2013.

[14] Heberle, F. A.; Petruzielo, R. S.; Pan, J.; Drazba, P.; Kučerka, N.; Standaert, R. F.; Feigenson, G. W.; Katsaras, J. *J. Am. Chem. Soc.* **2013**, *135*, 6853–6859.

[15] Jiang, F. Y.; Bouret, Y.; Kindt, J. T. *Biophys. J.* **2004**, *87*, 182–192.

[16] Veatch, S. L.; Keller, S. L. *Biophys. J.* **2003**, *85*, 3074–3083.

[17] Klump, H. H.; Gaber, B. P.; Peticolas, W. L.; Yager, P. *Thermochim. Acta* **1981**, *48*, 361–366.

[18] Kučerka, N.; Nieh, M.-P.; Katsaras, J. *BBA-Biomembranes* **2011**, *1808*, 2761–2771.

[19] Nagle, J. F.; Tristram-Nagle, S. *BBA-Rev. Biomembranes* **2000**, *1469*, 159–195.

[20] Sun, W.; Tristram-Nagle, S.; Suter, R.; Nagle, J. *Biophys. J.* **1996**, *71*, 885–891.

[21] Douliez, J.-P.; Leonard, A.; Dufourc, E. J. *Biophys. J.* **1995**, *68*, 1727–1739.

[22] Wu, E.; Jacobson, K.; Papahadjopoulos, D. *Biochemistry* **1977**, *16*, 3936–3941.

[23] Brulet, P.; McConnell, H. M. *Proc. Natl. Acad. Sci. USA* **1975**, *72*, 1451–1455.

[24] Cullis, P. *FEBS Lett.* **1976**, *70*, 223–228.

[25] Meyer, R.; Sonnen, A. F.-P.; Nau, W. M. *Langmuir* **2010**, *26*, 14723–14729.

[26] Chiu, S.; Jakobsson, E.; Mashl, R. J.; Scott, H. L. *Biophys. J.* **2002**, *83*, 1842–1853.

[27] Hofsäß, C.; Lindahl, E.; Edholm, O. *Biophys. J.* **2003**, *84*, 2192–2206.

[28] Edholm, O.; Nagle, J. F. *Biophys. J.* **2005**, *89*, 1827–1832.

[29] Sankaram, M. B.; Thompson, T. E. *Biochemistry* **1990**, *29*, 10676–10684.

[30] Reinl, H.; Brumm, T.; Bayerl, T. M. *Biophys. J.* **1992**, *61*, 1025.

[31] Leonenko, Z.; Finot, E.; Ma, H.; Dahms, T.; Cramb, D. *Biophys. J.* **2004**, *86*, 3783–3793.

[32] Sodt, A. J.; Sandar, M. L.; Gawrisch, K.; Pastor, R. W.; Lyman, E. *J. Am. Chem. Soc.* **2014**, *136*, 725–732.

[33] Clarke, J. A.; Heron, A. J.; Seddon, J. M.; Law, R. V. *Biophys. J.* **2006**, *90*, 2383–2393.

[34] Tian, A.; Johnson, C.; Wang, W.; Baumgart, T. *Phys. Rev. Lett.* **2007**, *98*, 208102.

[35] Blanchette, C. D.; Lin, W.-C.; Orme, C. A.; Ratto, T. V.; Longo, M. L. *Langmuir* **2007**, *23*, 5875–5877.

[36] Schäfer, L. V.; Marrink, S. J. *Biophys. J.* **2010**, *99*, L91–L93.

[37] Putzel, G. G.; Uline, M. J.; Szleifer, I.; Schick, M. *Biophys. J.* **2011**, *100*, 996–1004.

[38] Bennett, W. D.; MacCallum, J. L.; Hinner, M. J.; Marrink, S. J.; Tieleman, D. P. *J. Am. Chem. Soc.* **2009**, *131*, 12714–12720.

[39] Hamilton, J. A. *Curr. Opin. Lipidol.* **2003**, *14*, 263–271.

[40] May, S. *Soft Matter* **2009**, *5*, 3148–3156.

[41] Goren, M. A.; Morizumi, T.; Menon, I.; Joseph, J. S.; Dittman, J. S.; Cherezov, V.; Stevens, R. C.; Ernst, O. P.; Menon, A. K. *Nat. Commun.* **2014**, *5*.

[42] Menon, I.; Huber, T.; Sanyal, S.; Banerjee, S.; Barré, P.; Canis, S.; Warren, J. D.; Hwa, J.; Sakmar, T. P.; Menon, A. K. *Curr. Biol.* **2011**, *21*, 149–153.

[43] Pöyry, S.; Róg, T.; Karttunen, M.; Vattulainen, I. *J. Phys. Chem. B* **2008**, *112*, 2922–2929.

[44] Krause, M. R.; Wang, M.; Mydock-McGrane, L.; Covey, D. F.; Tejada, E.; Almeida, P. F.; Regen, S. L. *Langmuir* **2014**,

[45] Waheed, Q.; Tjörnhammar, R.; Edholm, O. *Biophys. J.* **2012**, *103*, 2125–2133.

[46] Zhang, Y.; Lervik, A.; Seddon, J.; Bresme, F. *Chem. Phys. Lipids* **2015**, *185*, 88–98.

[47] Wang, Y.; Gkeka, P.; Fuchs, J. E.; Liedl, K. R.; Cournia, Z. *BBA-Biomembranes* **2016**, *1858*, 2846–2857.

[48] Pandit, S. A.; Khelashvili, G.; Jakobsson, E.; Grama, A.; Scott, H. *Biophys. J.* **2007**, *92*, 440–447.

[49] Tjörnhammar, R.; Edholm, O. *J. Chem. Theory Comput.* **2014**, *10*, 5706–5715.
